# Supplementary material for: A general reaction mechanism for carbapenem hydrolysis by mononuclear and binuclear metallo-β-lactamases
Source: Nat Commun. 2017 Sep 14;8:538. doi: 10.1038/s41467-017-00601-9 (PMC5599593; doi:10.1038/s41467-017-00601-9)
Supplement: Supplementary file 1 — Supplementary Information [file 41467_2017_601_MOESM1_ESM.pdf]

Description of Supplementary Files

File name: Supplementary Information
Description: Supplementary figures, supplementary tables and supplementary references.

File name: Peer review file

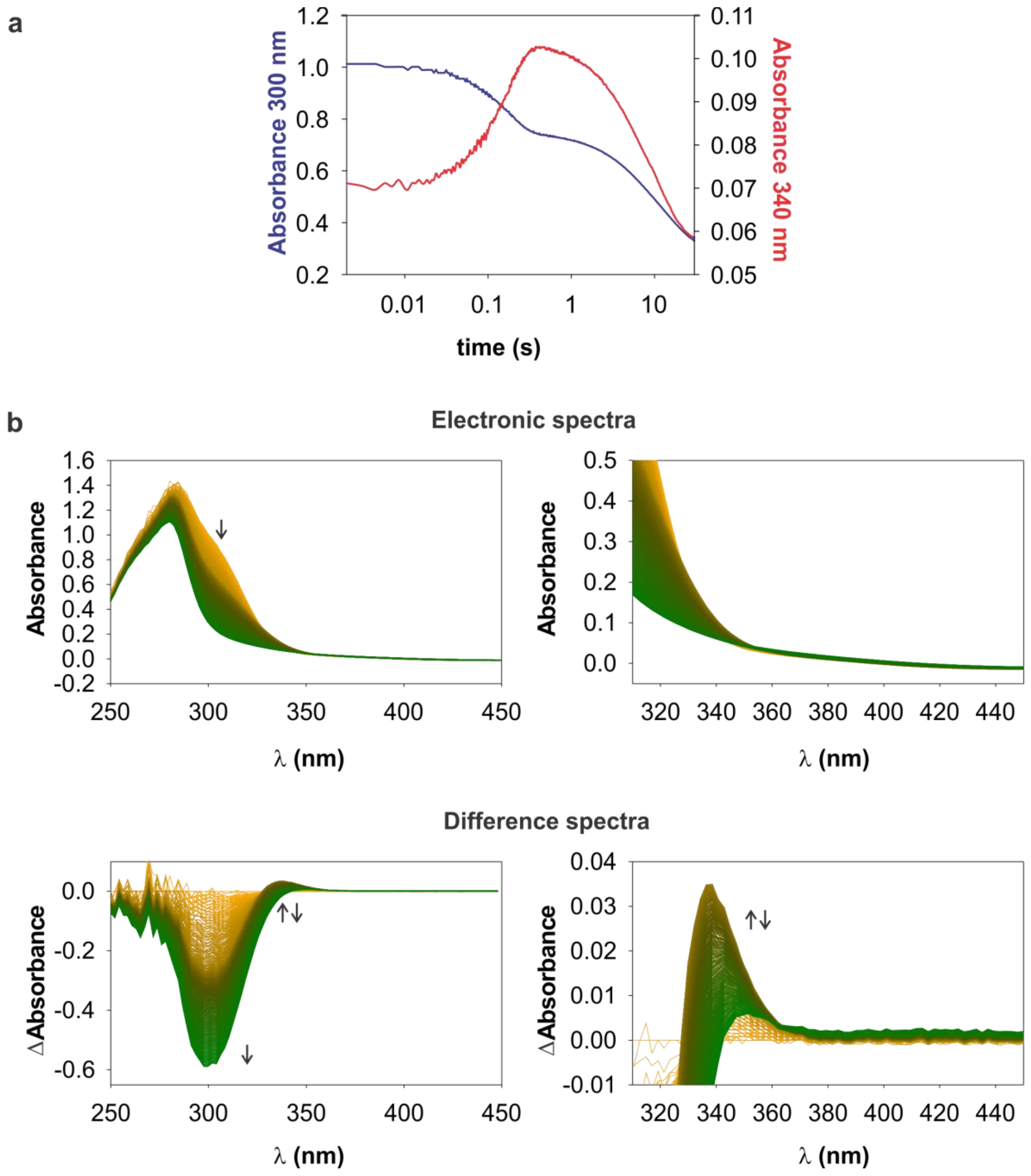

Supplementary Figure 1 | Electronic absorption spectra of imipenem hydrolysis catalyzed by mono-Zn(II)-GOB-18 under pre-steady state conditions. Hydrolysis of 91 µM imipenem by 51.4 µM mono-Zn(II)-GOB-18 detected employing a stopped-flow mixer coupled to a photodiode array detector. 500 spectra were recorded in 50 s, with a logarithmic time base. The measurement was performed in 100 mM Hepes, pH 7.5, 200 mM NaCl, at 4 ºC. (**a**) Absorbance evolution in the course of the reaction at 300 nm (blue) and 340 nm (red). (**b**) Upper panels: sequence of electronic spectra. Lower panels: sequence of difference spectra obtained by subtraction of the first spectrum recorded. The reaction progresses from yellow to green spectra.

a

$$E + S \underset{k_{-1}}{\overset{k_{+1}}{\rightleftharpoons}} ES \xrightarrow{k_2} EI^1 \xrightarrow{k_3} EP \underset{k_{-4}}{\overset{k_{+4}}{\rightleftharpoons}} E + P$$

b

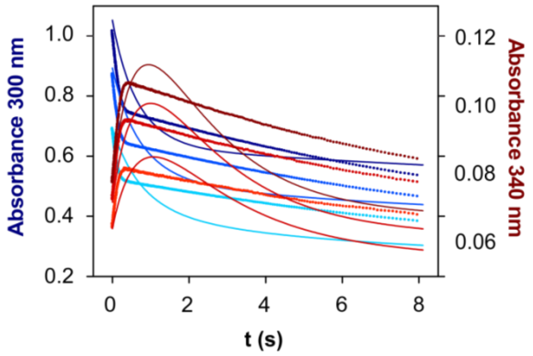

k_{+1}	ND
k_{-1}	ND
k_2	ND
k_3	(0.83 ± 0.03) s^{-1}
k_{+4}	ND
k_{-4}	ND
$\varepsilon_{EI\ 340\ nm}$	(3,300 ± 70) M^{-1} cm^{-1}

c

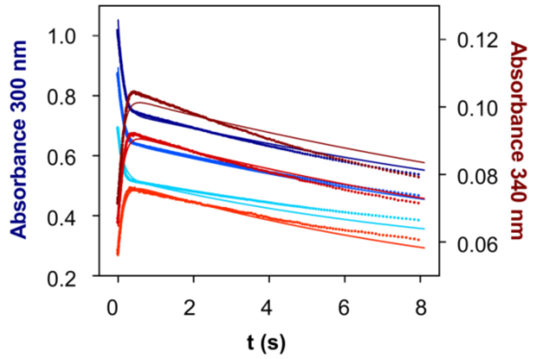

k_{+1}	(17 ± 6) s^{-1} μM^{-1}
k_{-1}	ND
k_2	(7.4 ± 0.1) s^{-1}
k_3	(0.1031 ± 0.0009) s^{-1}
k_{+4}	(1.2 ± 0.5) s^{-1}
k_{-4}	(300 ± 200) s^{-1} μM^{-1}
$\varepsilon_{EI\ 300\ nm}$	(8,460 ± 20) M^{-1} cm^{-1}
$\varepsilon_{EI\ 340\ nm}$	(1,606 ± 4) M^{-1} cm^{-1}

d

$$E + S \underset{k_{-1}}{\overset{k_{+1}}{\rightleftharpoons}} ES \xrightarrow{k_2} EI^1 \xrightarrow{k_3} EI^2 \xrightarrow{k_4} EP \underset{k_{-5}}{\overset{k_{+5}}{\rightleftharpoons}} E + P$$

e

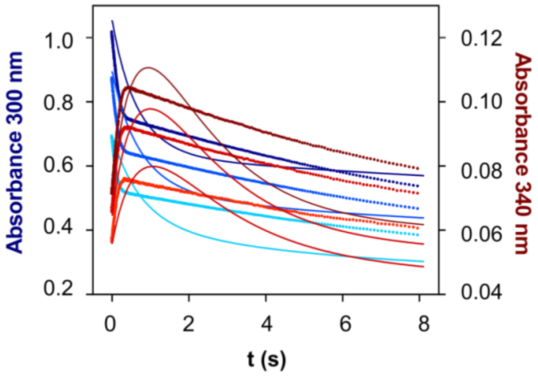

k_1	ND
k_{-1}	ND
k_2	ND
k_3	ND
k_4	(0.86 ± 0.03) s^{-1}
k_{+5}	ND
k_{-5}	ND
$\varepsilon_{EI2\ 340\ nm}$	(3,330 ± 80) M^{-1} cm^{-1}

f

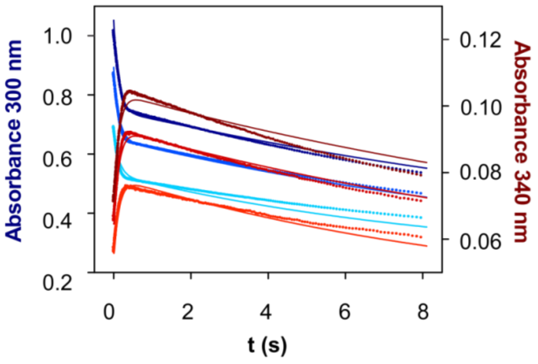

k_{+1}	(13 ± 3) s^{-1} μM^{-1}
k_{-1}	ND
k_2	(7.38 ± 0.09) s^{-1}
k_3	(159 ± 8) s^{-1}
k_4	(0.1073 ± 0.0009) s^{-1}
k_{+5}	(1.4 ± 0.7) s^{-1}
k_{-5}	(300 ± 200) s^{-1} μM^{-1}
$\varepsilon_{EI2\ 300\ nm}$	(8,517 ± 20) M^{-1} cm^{-1}
$\varepsilon_{EI2\ 340\ nm}$	(1,617 ± 3) M^{-1} cm^{-1}

Supplementary Figure 2 | Global fit of traces of imipenem hydrolysis catalyzed by mono-Zn(II)-GOB-18 under pre-steady state conditions.

g

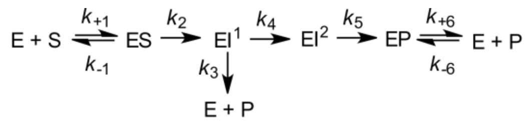

h

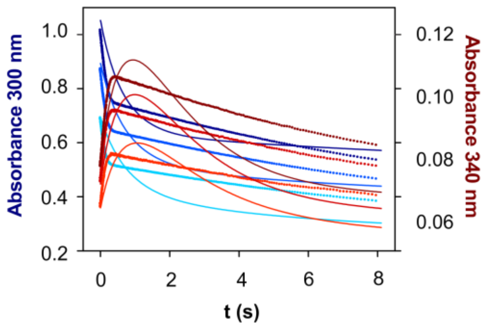

k_{+1}	ND
k_{-1}	ND
k_2	ND
k_3	ND
k_4	ND
k_5	$(0.86 \pm 0.03)\ s^{-1}$
k_{+6}	ND
k_{-6}	ND
$\varepsilon_{EI2\ 340\ nm}$	$(3{,}330 \pm 80)\ M^{-1}\ cm^{-1}$

i

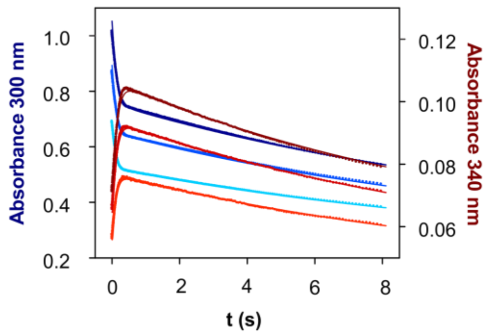

k_{+1}	$(1.6 \pm 0.1)\ s^{-1}\ \mu M^{-1}$
k_{-1}	$(2.8 \pm 0.8)\ s^{-1}$
k_2	$(12.6 \pm 0.4)\ s^{-1}$
k_3	$(132 \pm 6)\ s^{-1}$
k_4	$(69 \pm 5)\ s^{-1}$
k_5	$(0.0812 \pm 0.0004)\ s^{-1}$
k_{+6}	ND
k_{-6}	$(0.43 \pm 0.07)\ s^{-1}\ \mu M^{-1}$
$\varepsilon_{EI2\ 300\ nm}$	$(20{,}000 \pm 1{,}000)\ M^{-1}\ cm^{-1}$
$\varepsilon_{EI2\ 340\ nm}$	$(2{,}700 \pm 100)\ M^{-1}\ cm^{-1}$

Supplementary Figure 2 cont. | Global fit of traces of imipenem hydrolysis catalyzed by mono-Zn(II)-GOB-18 under pre-steady state conditions. (**a**) Scheme that describes a linear mechanism, which includes a transient species (EI^1) and the complexes of the enzyme (E) with the substrate (ES) and the product (EP). (**b,c**) Fits to the mechanism shown in (a), assuming that EI^1 only absorbs at 340 nm (b) or EI^1 absorbs both at 300 and 340 nm (c). In both cases, fits were qualitatively and quantitatively deficient, and the models did not reproduce the empirical data. (**d**) Scheme that describes a linear mechanism, which includes two transient species (EI^1 and EI^2) (**e,f**) Fits to the mechanism shown in (d). We reasoned that EI^1 might be consumed during the dead time of the experiment or that it could accumulate at low concentrations and. Besides, we considered that only the intermediate EI^2 contributed to the absorbance at 340 nm. (e) Fit assuming that EI^2 did not absorb at 300 nm. (f) Fit allowing the program to estimate a value for $\varepsilon_{EI2\ 300\ nm}$. In both cases, fits were deficient which evidenced the necessity of adding further steps in the kinetic scheme (**g**) Scheme that describes a branched mechanism, which includes two transient and productive species (EI^1 and EI^2). (**h,i**) Fits to the mechanism shown in (g), assuming that EI^2 only absorbs at 340 nm (h) or that EI^2 absorbs both at 300 and 340 nm (i). Fit (i) showed that model (g) was sufficient to reproduce the experimental data if a spectral contribution was assigned to EI^2 at 300 nm.

In all cases, evolution of the absorbance at 300nm is shown in blue and at 340 nm in red and correspond to the reaction of 52.6 μM imipenem and 51.4 μM enzyme (light colours); 74.0 μM

imipenem and 51.4 μM enzyme (medium tones colours) and 91.0 μM imipenem and 51.4 μM enzyme (dark colours). Measurements were performed in 100 mM Hepes, pH 7.5, 200 mM NaCl, at 4º C. Traces resulting from the fits to different kinetic schemes (lines) are shown overlaid with the experimental data (dots). The parameters derived by analysis with DynaFit[1] are shown at the right in each case. Fixed absorption coefficients are detailed in Materials and Methods. ND: not defined value.

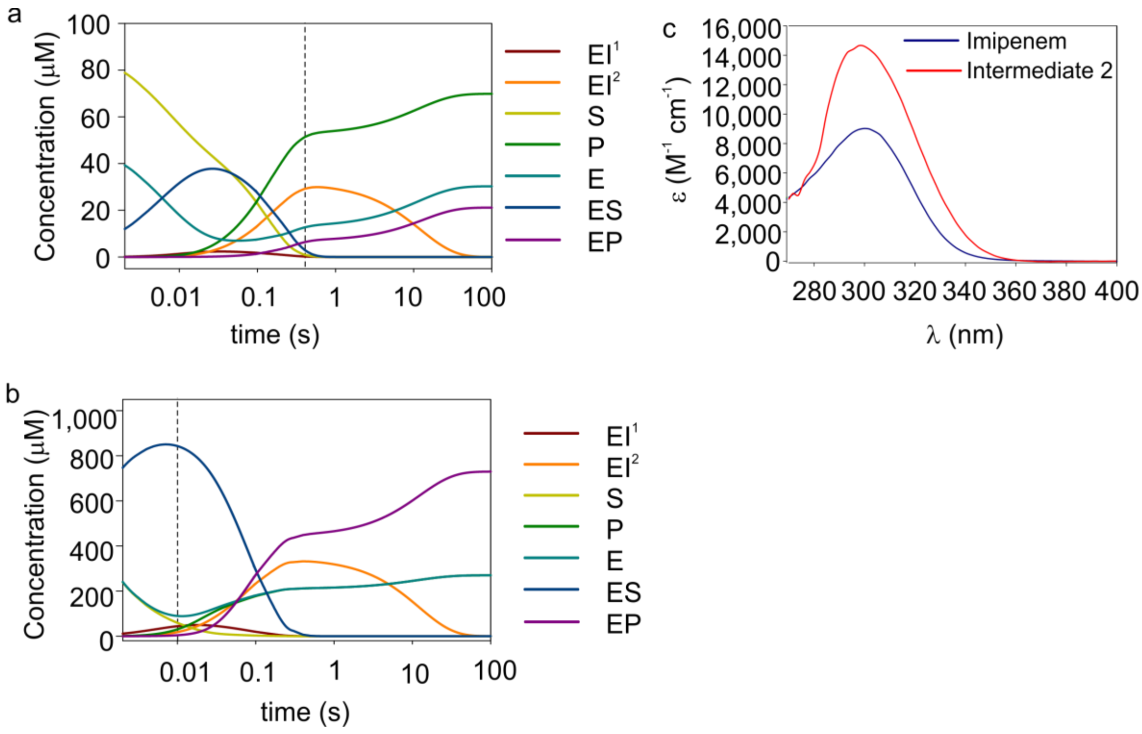

Supplementary Figure 3 | Species accumulation during imipenem hydrolysis by mono-Zn(II)-GOB-18. (**a**) Species concentrations during the reaction of 91 µM imipenem and 51.4 µM mono-Zn(II)-GOB-18 (stopped flow experiments conditions). The species simulation was performed with DynaFit[1] based on the mechanistic model and the kinetic parameters shown in Supplementary Fig. 2.i. The black dashed line indicates the maximum accumulation of EI^2 at 600 ms. As k_{+6} could not be fitted, it was fixed for the simulation at 100 times k_{-6}, as was observed for the fit of mono-Co(II)-GOB-18. Different values of k_{+6} did not affect accumulation of ES, EI^1 or EI^2. (**b**) Species concentration during the reaction of 1mM mono-Zn(II)-GOB-18 and 1 mM imipenem (EXAFS conditions). The black dashed line shows that ES is the predominant species at 10 ms of reaction. As k_{+6} could not be fitted, it was fixed for the simulations at 100 times k_{-6}, as was observed for the fit of mono-Co(II)-GOB-18. As k_{+6} could not be fitted, it was fixed for the simulation at 100 times k_{-6}, as was observed for the fit of mono-Co(II)-GOB-18. Different values of k_{+6} did not affect accumulation of ES, EI^1 or EI^2. (**c**) Spectrum of Intermediate 2 (red) accumulated during imipenem hydrolysis by mono-Zn(II)-GOB-18, compared to the spectrum of imipenem (blue). The spectrum of Intermediate 2 was obtained by subtraction of the spectra of free enzyme and of substrate from the spectrum recorded after 600 ms of reaction of 91 µM imipenem and 51.4 µM mono-Zn(II)-GOB-18, when EI^2 is the major species. The difference spectrum displayed an absorption band with a maximum at 297 nm (λ_{max}) and a shoulder at 340 nm. Based on the species simulation, the concentrations of the different species at that time were: 29.9 µM EI^2, 0.03 µM EI^1, 13.7 µM E, 7.3 µM EP, 0.45 µM ES, 0.11 µM S, 53.2 µM P.

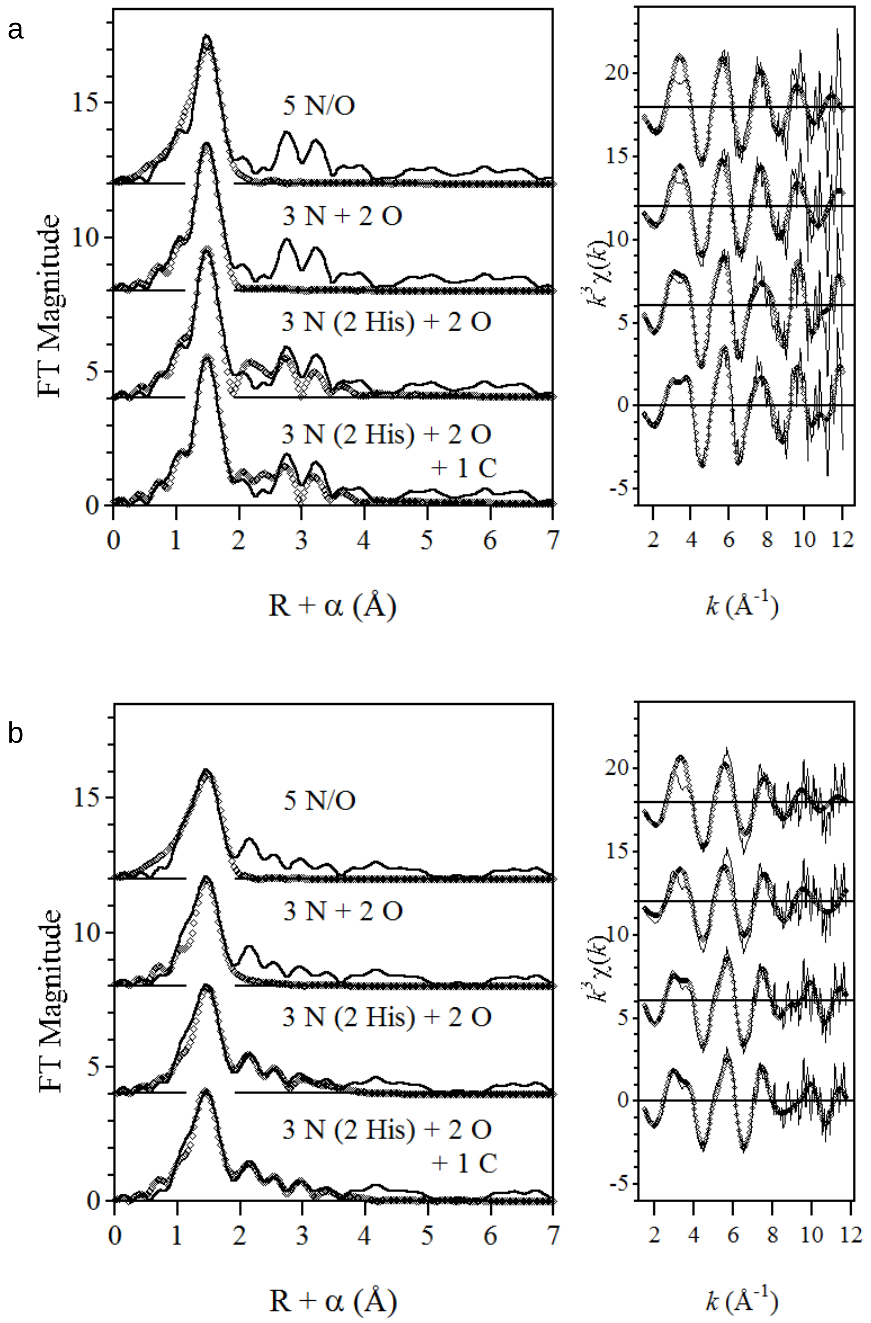

Supplementary Figure 4 | Fourier transforms of k^3-weighted EXAFS for mono-Zn(II)-GOB-18 reaction. (**a**) Fourier transforms (left) of k^3-weighted EXAFS (right) for mono-Zn(II)-GOB-18 freeze-quenched after 10 ms of reaction with imipenem (solid lines), and corresponding curve fits (open symbols). The outer shell scattering showed a dramatic increase in the Fourier transform (FT) intensity at R + α ~ 2.7 and 3.3 Å in the 10 ms data, which relaxed substantially in the EP complex. (**b**) Fourier transforms (left) of k^3-weighted EXAFS (right) for the GOB-imipenem product complex (solid lines), and corresponding curve fits (open symbols). Supplementary Table 2 shows detailed EXAFS curve fitting results.

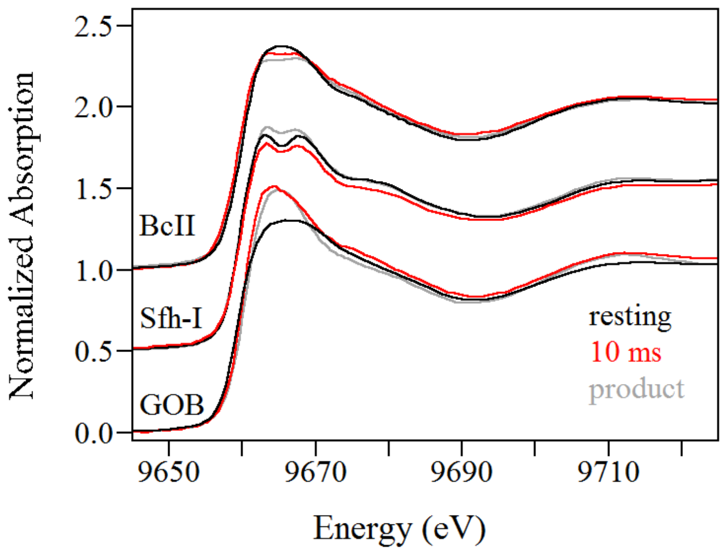

Supplementary Figure 5 | Normalized XANES. Bi-Zn(II)-BcII (top), mono-Zn(II)-Sfh-I (center) and mono-Zn(II)-GOB-18 (bottom) in the resting state (black line), freeze-quenched after 10 ms of reaction with imipenem (red lines) and the corresponding product complexes (gray lines).

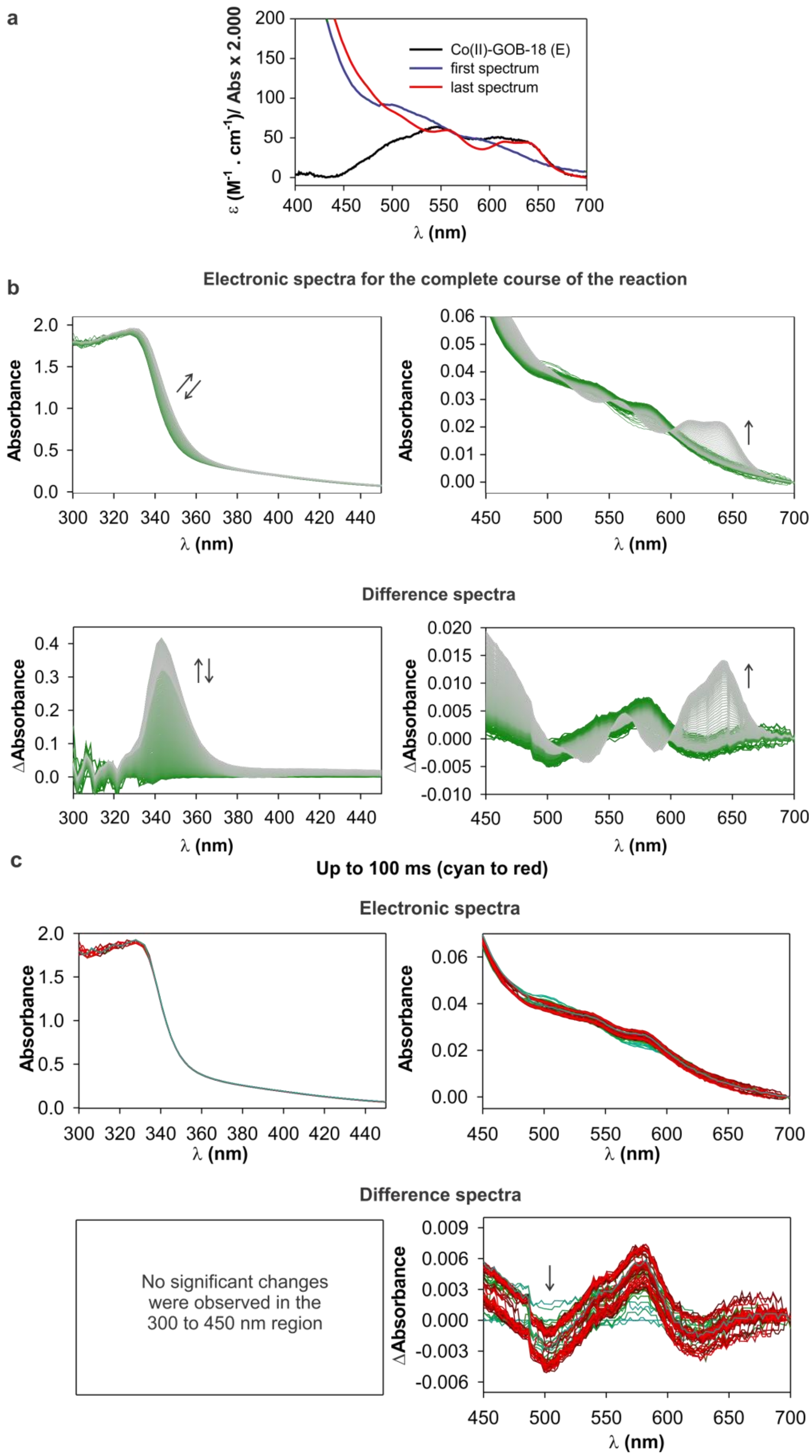

Supplementary Figure 6 | Electronic absorption spectra of imipenem hydrolysis catalyzed by mono-Co(II)-GOB-18 under pre-steady state conditions.

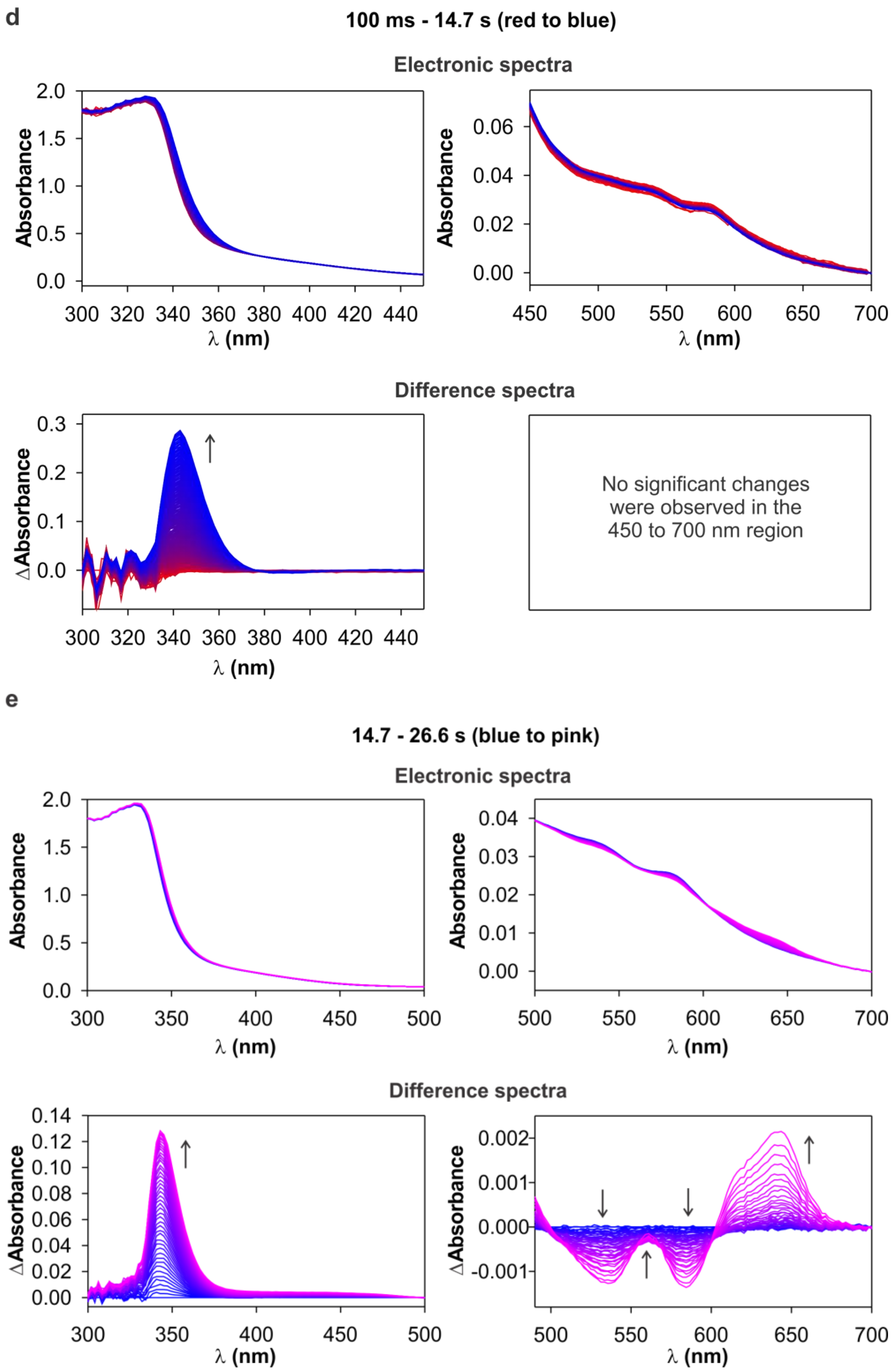

Supplementary Figure 6 cont.| Electronic absorption spectra of imipenem hydrolysis catalyzed by mono-Co(II)-GOB-18 under pre-steady state conditions.

f

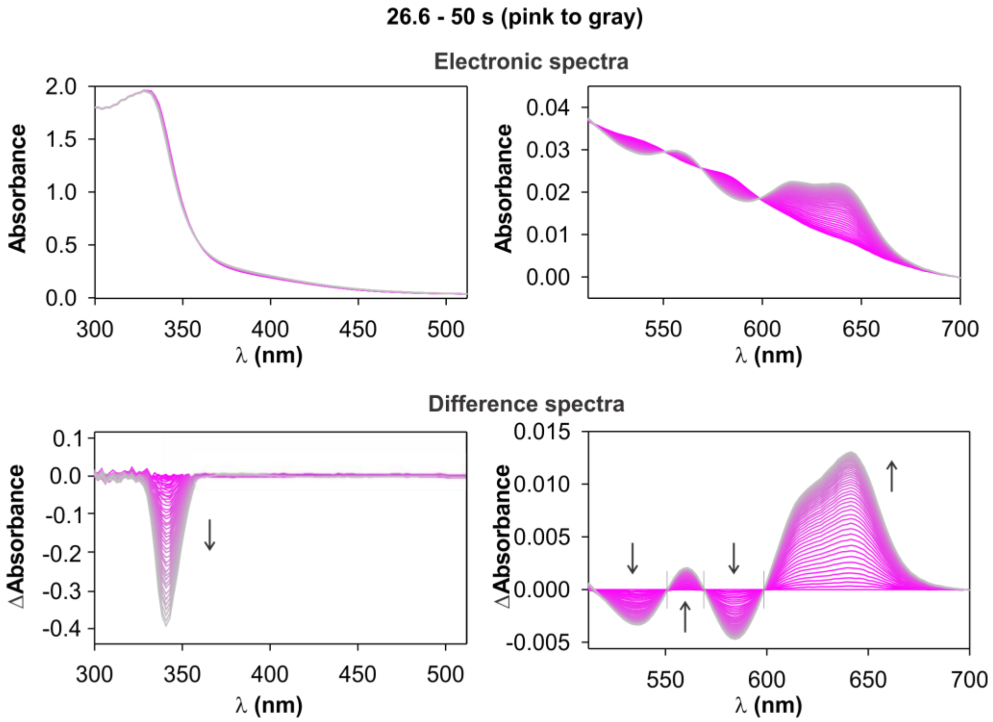

Supplementary Figure 6 cont.| Electronic absorption spectra of imipenem hydrolysis catalyzed by mono-Co(II)-GOB-18 under pre-steady state conditions. Hydrolysis of 3.5 mM imipenem by 430 μM mono-Co(II)-GOB-18 detected employing a stopped-flow mixer coupled to a photodiode array detector. 500 spectra were recorded in 50 s, with a logarithmic time base. The measurement was performed in 100 mM Hepes, pH 7.5, 200 mM NaCl, at 4 ºC. (**a**) Comparison of the first and last spectra obtained upon the reaction and spectrum of free mono-Co(II)-GOB-18. (**b**) Electronic spectra of the complete course of the reaction. The reaction progresses from green to grey spectra. Upper panels: sequence of electronic spectra. Lower panels: sequence of difference spectra obtained by subtraction of the first spectrum recorded. Changes in the d-d bands were clearly observed (450-700 nm), revealing the accumulation and later decay of an intermediate with absorbance at 340 nm, identical to what was found for mono-Zn(II)-GOB-18. (**c**) Electronic spectra recorded up to 100 ms of reaction. The reaction progresses from cyan to red spectra. Upper panels: sequence of electronic spectra. Lower panel: sequence of difference spectra obtained by subtraction of the first spectrum recorded. The spectrum of free mono-Co(II)-GOB-18 disappeared and a new species was formed, giving rise to different d-d bands and isosbestic points at 475, 525 and 600 nm, approximately. (**d**) Electronic spectra recorded during 100 ms to 14.7 s of reaction. The reaction progresses from red to blue spectra. Upper panels: sequence of electronic spectra. Lower panel: sequence of difference spectra obtained by subtraction of the spectrum recorded at 100 ms. A species with absorbance at 340 nm started accumulating without changes in the ligand field bands up to 100 ms of reaction. (**e**) Electronic spectra recorded during 14.7 to 26.6 s of reaction. The reaction progresses from blue to pink spectra. Upper panels: sequence of electronic spectra. Lower panels: sequence of difference spectra obtained by subtraction of the spectrum recorded at

14.7 s. The intensity of the band at 340 nm continued rising and the d-d bands of free enzyme began to recover. (**f**) Electronic spectra recorded during 26.6 to 50 s of reaction. The reaction progresses from pink to grey spectra. Upper panels: sequence of electronic spectra. Lower panels: sequence of difference spectra obtained by subtraction of the spectrum recorded at 26.6 s. The species that absorbs at 340 nm decayed and the d-d bands of free enzyme are recovered, with isosbestic points at 550, 570 and 600 nm.

a

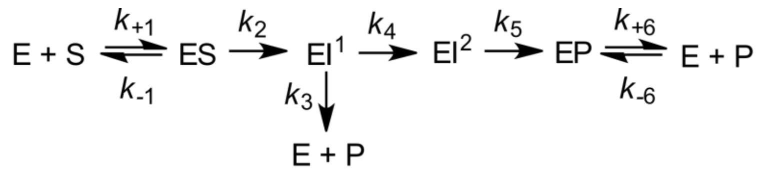

b

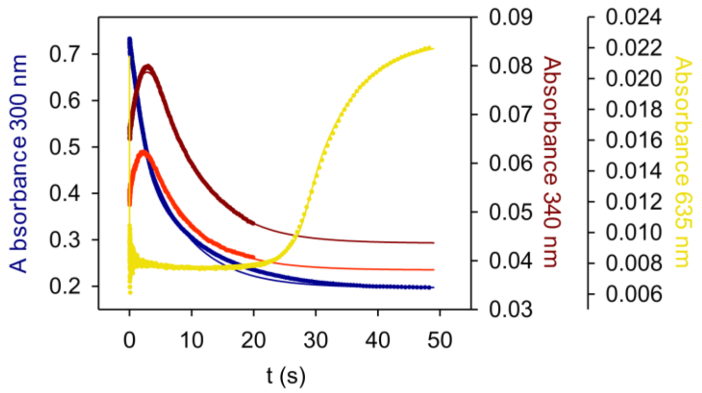

c

k_1	$(0.32 \pm 0.02)\ s^{-1}\ \mu M^{-1}$	$\varepsilon_{EI2\ 300\ nm}$	$(19{,}000 \pm 2{,}000)\ M^{-1}\ cm^{-1}$
k_{-1}	ND	$\varepsilon_{EI2\ 340\ nm}$	$(2{,}100 \pm 200)\ M^{-1}\ cm^{-1}$
k_2	$(0.97 \pm 0.02)\ s^{-1}$	$\varepsilon_{E\ 635\ nm}$	$50\ M^{-1}\ cm^{-1}$ (fixed)
k_3	$(24 \pm 3)\ s^{-1}$	$\varepsilon_{ES\ 635\ nm}$	$(19.80 \pm 0.08)\ M^{-1}\ cm^{-1}$
k_4	$(16 \pm 2)\ s^{-1}$	$\varepsilon_{EI1\ 635\ nm}$	$16.7\ M^{-1}\ cm^{-1}$ (fixed)
k_5	$(0.141 \pm 0.001)\ s^{-1}$	$\varepsilon_{EI2\ 635\ nm}$	$(15.5 \pm 0.7)\ M^{-1}\ cm^{-1}$
k_6	$(3 \pm 1)\ s^{-1}$	$\varepsilon_{EP\ 635\ nm}$	$(52.5 \pm 0.2)\ M^{-1}\ cm^{-1}$
k_{-6}	$(0.03 \pm 0.01)\ s^{-1}\ \mu M^{-1}$		

Supplementary Figure 7 | Global fit of traces of imipenem hydrolysis catalyzed by mono-Co(II)-GOB-18 under pre-steady state conditions. (**a**) Scheme that describes the kinetic model which allowed the best fit of the experimental data after an analysis similar to that described in Supplementary Fig. 2. It includes two transient and productive species (EI^1 and EI^2, the second of them presenting a spectral contribution at 340 nm) and the complexes of the enzyme (E) with the substrate (ES) and the product (EP). (**b**) Evolution of the absorbance at 300 (blue), 340 (red) and 635 nm (yellow) upon the reaction of: i) 60.0 μM imipenem and 42.8 μM mono-Co(II)-GOB-18 (blue and light red dots); ii) 95.0 μM imipenem and 42.8 μM mono-Co(II)-GOB-18 (dark red dots) and iii) 3.5 mM imipenem and 430 μM mono-Co(II)-GOB-18 (green dots). The measurements were performed in 100 mM Hepes pH 7.5, 200 mM NaCl, at 4º C. The results of the fits to the kinetic model in (a) are shown as lines overlaid with the experimental data (dots). Notably, the model was also capable of reproducing the evolution of the absorbance at 635 nm, which corresponds to the position of one of the absorption maxima of the free enzyme (see Supplementary Fig. 6a). The molar extinction coefficients estimated for the complexes ES and EI^2 were consistent with an expansion of the coordination sphere upon reaction (**c**) Parameters derived from the fit with the program DynaFit[1]. Fixed absorption coefficients are detailed in Materials and Methods. ND: not defined value.

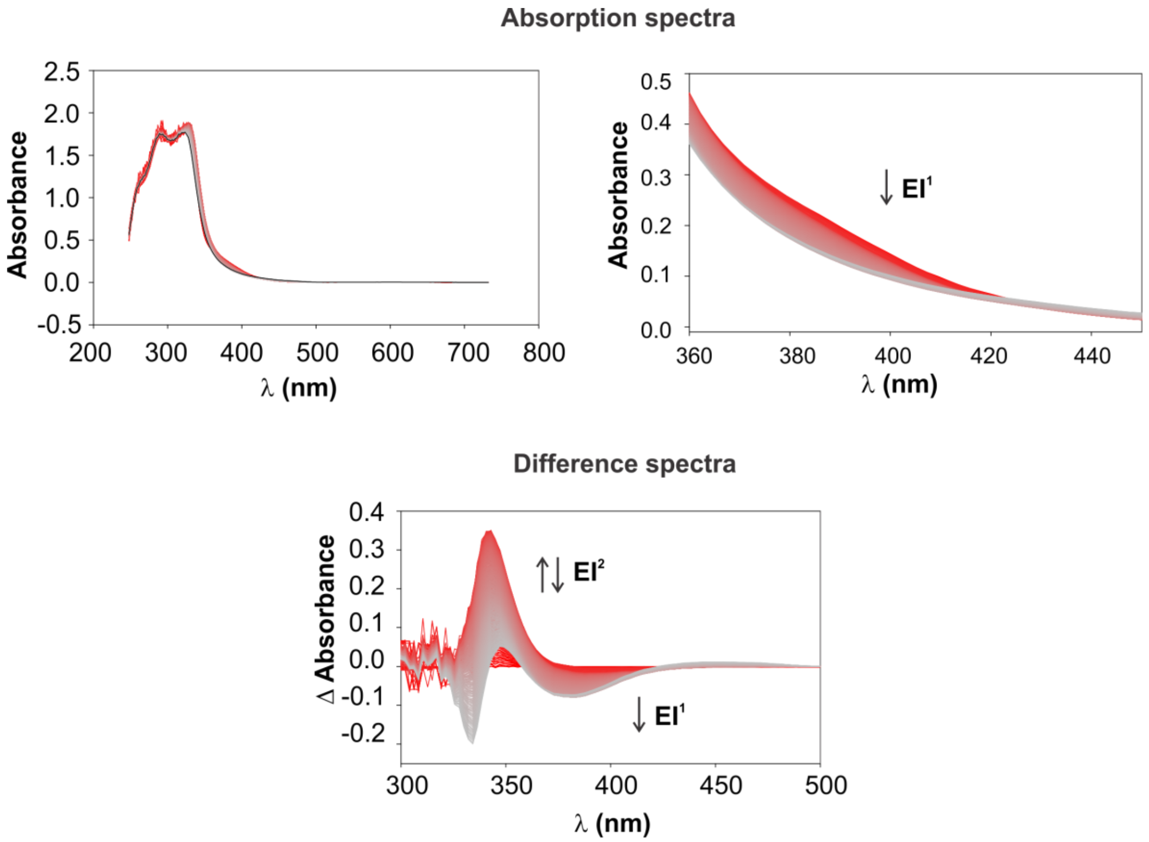

Supplementary Figure 8 | Electronic absorption spectra of imipenem hydrolysis catalyzed by mono-Zn(II)-Sfh-I under pre-steady state conditions. Hydrolysis of 3 mM imipenem by 500 µM mono-Zn(II)-Sfh-I detected employing a stopped-flow mixer coupled to a photodiode array detector. 500 spectra were recorded in 50 s, with a logarithmic time base. The measurement was performed in 50 mM Hepes, pH 7, at 4 ºC. Upper panels: sequence of electronic spectra of the complete course of the reaction. Lower panel: sequence of differential spectra corresponding to the data presented in the upper panel (obtained by subtraction of the first spectrum recorded). Two transient species were detected: EI^1 and EI^2. The accumulation of EI^1 was evidenced in the early steps of the reaction by its absorbance at 390 nm. EI^2 accumulated later, showing a maximum of differential absorption at 340 nm. The reaction progresses from red to grey spectra.

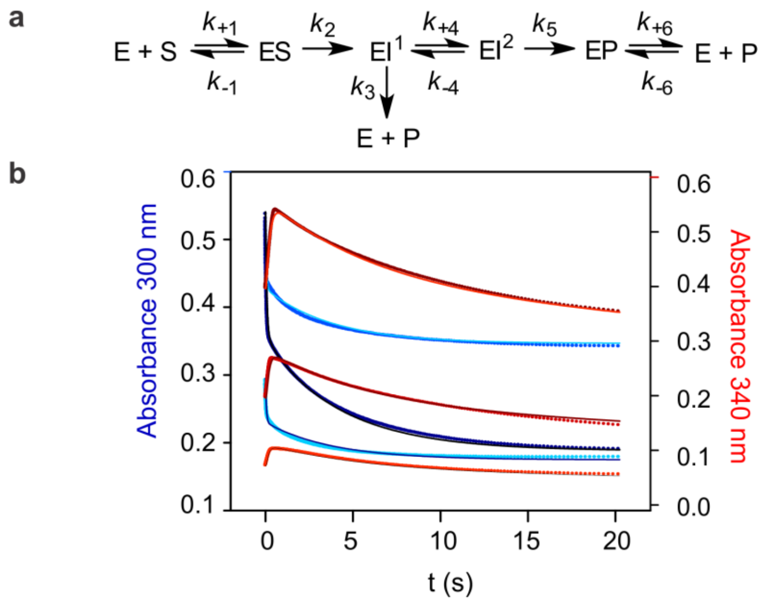

c

k_{+1}	$(24 \pm 3)\ s^{-1}\ \mu M^{-1}$	k_5	$(0.09 \pm 0.03)\ s^{-1}$
k_{-1}	$(16 \pm 5)\ s^{-1}$	k_{+6}	$(0.39 \pm 0.05)\ s^{-1}$
k_2	$(12.5 \pm 0.1)\ s^{-1}$	k_{-6}	$(60 \pm 10)\ s^{-1}$
k_3	$(19.0 \pm 0.8)\ s^{-1}$	$\varepsilon_{EI2\ 300\ nm}$	$(21{,}000 \pm 2{,}000)\ M^{-1}\ cm^{-1}$
k_{+4}	$(5.8 \pm 0.6)\ s^{-1}$	$\varepsilon_{EI2\ 340\ nm}$	$(5{,}000 \pm 1{,}000)\ M^{-1}\ cm^{-1}$
k_{-4}	$(0.22 \pm 0.05)\ s^{-1}$		

Supplementary Figure 9 | Global fit of traces of imipenem hydrolysis catalyzed by mono-Zn(II)-Sfh-I under pre-steady state conditions. (**a**) Scheme that describes the kinetic model which allowed the best fit of the experimental data after an analysis similar to that described in Supplementary Fig. 2. It includes two transient and productive species (EI^1 and EI^2, the second of them presenting a spectral contribution at 340 nm) and the complexes of the enzyme (E) with the substrate (ES) and the product (EP). (**b**) Evolution of the absorbance at 300 and 340 nm upon the reaction of 13.6 μM imipenem and 30 μM mono-Zn(II)-Sfh-I (light blue dots); 21 μM imipenem and 60 μM mono-Zn(II)-Sfh-I (blue dots); 39.8 μM imipenem and 30 μM mono-Zn(II)-Sfh-I (dark blue dots); 152 μM imipenem and 30 μM mono-Zn(II)-Sfh-I (light red dots); 430 μM imipenem and 60 μM mono-Zn(II)-Sfh-I (red dots) and 916 μM imipenem and 60 μM mono-Zn(II)-Sfh-I (dark red dots). The measurements were performed in 50 mM Hepes pH 7, at 4º C. The results of the fits to the kinetic model in (a) are shown as lines overlaid with the experimental data (dots). Analysis was made with data obtained through 20 s of reaction. The program was allowed to estimate a value for $\varepsilon_{EI2\ 300\ nm}$. (**c**) Parameters derived from the fit with the program DynaFit[1]. Fixed absorption coefficients are detailed in Materials and Methods. ND: not defined value.

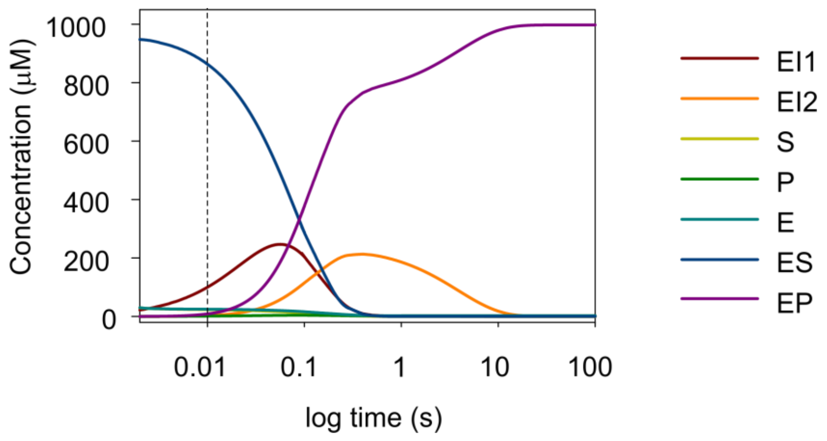

Supplementary Figure 10 | Species accumulation during imipenem hydrolysis by mono-Zn(II)-Sfh-I. Species concentration during the reaction of 1 mM imipenem and 1 mM mono-Zn(II)-Sfh-I (EXAFS conditions). The species simulation was performed with DynaFit[1] based on the mechanistic model presented on Supplementary Fig. 9a and using the kinetic constants displayed in the Supplementary Fig. 9c. The black dashed line shows that ES is the predominant species at 10 ms of reaction.

a

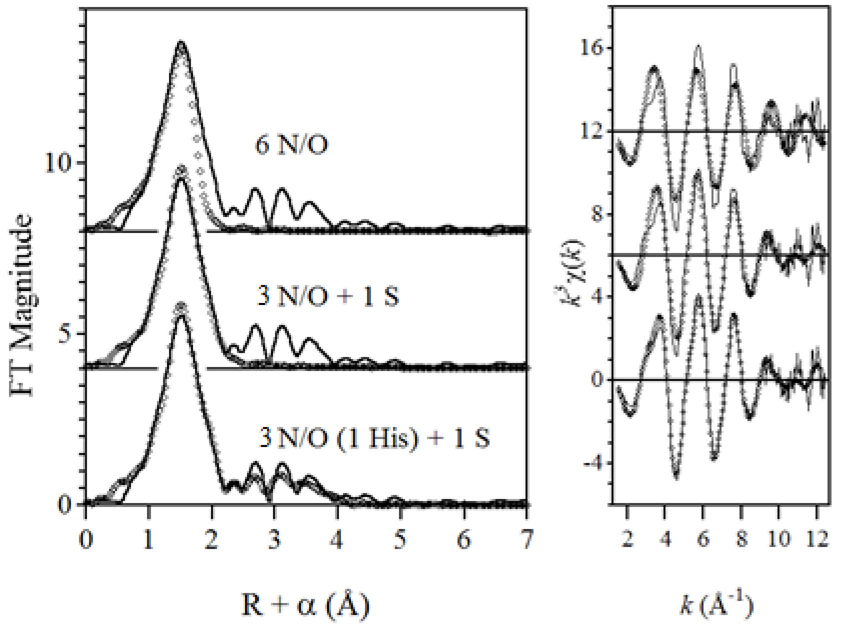

b

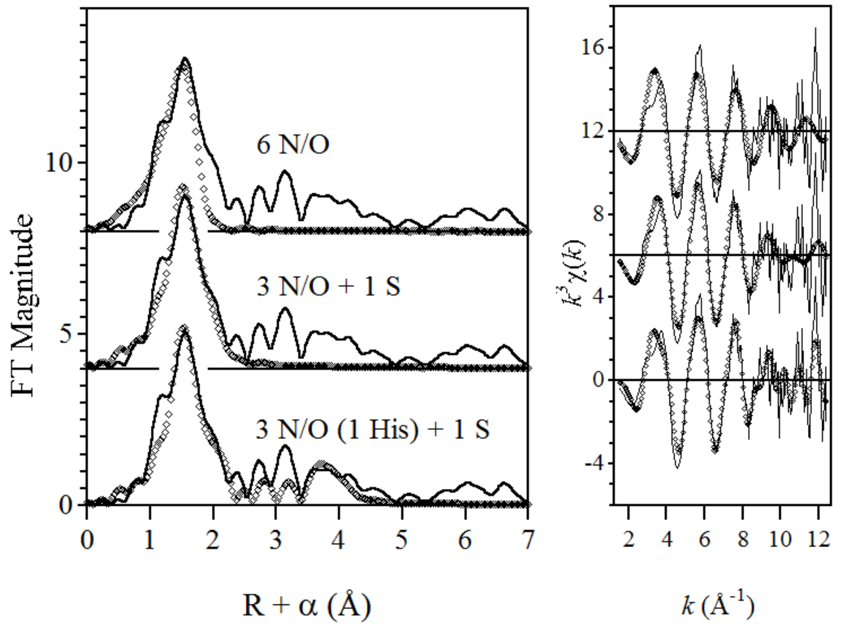

Supplementary Figure 11 | Fourier transforms of k^3-weighted EXAFS for mono-Zn(II)-Sfh-I reaction.

c

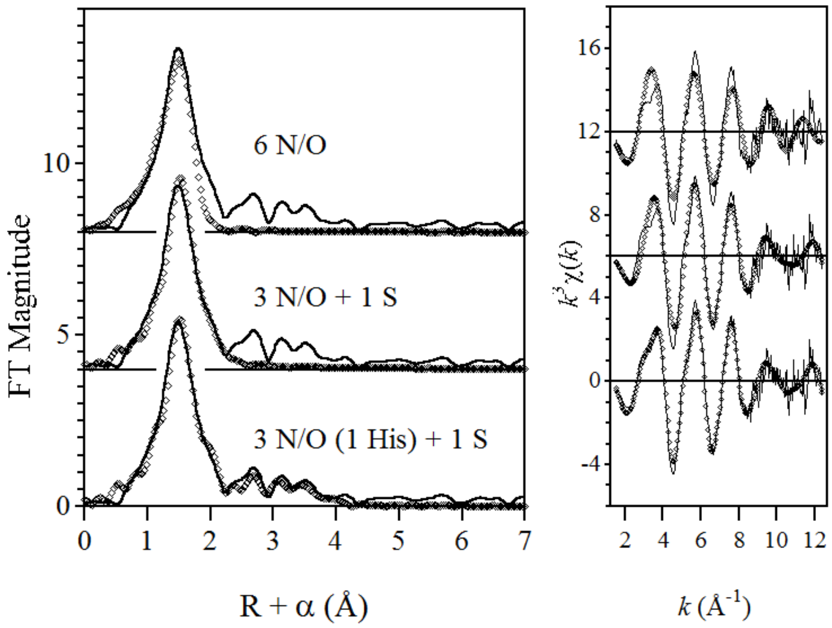

d

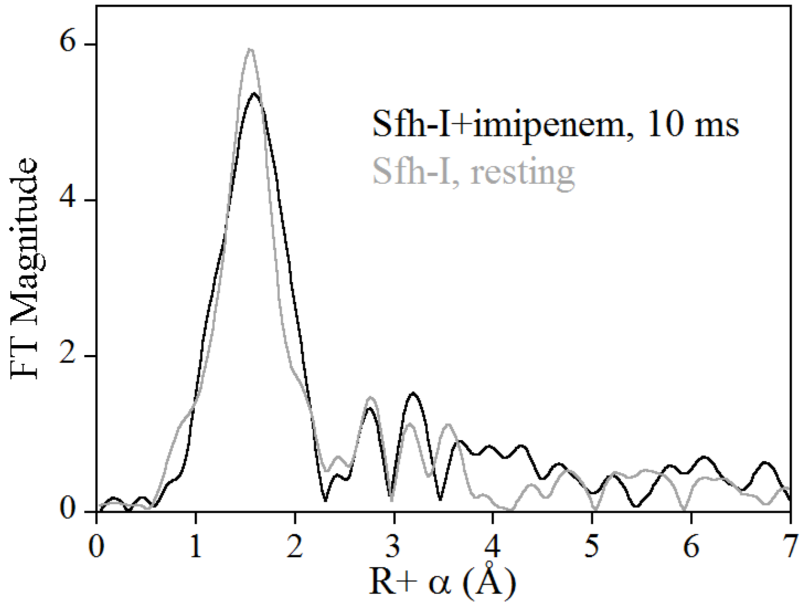

Supplementary Figure 11 cont. | Fourier transforms of k^3-weighted EXAFS for mono-Zn(II)-Sfh-I reaction. (**a**) Fourier transforms (left) of k^3-weighted EXAFS (right) for resting mono-Zn(II)-Sfh-I (solid lines), and corresponding curve fits (open symbols). (**b**) Fourier transforms (left) of k^3-weighted EXAFS (right) for mono-Zn(II)-Sfh-I freeze-quenched after 10 ms of reaction with imipenem (solid lines), and corresponding curve fits (open symbols). Supplementary Table 4 shows detailed EXAFS curve fitting results. (**c**) Fourier transforms (left) of k^3-weighted EXAFS (right) for mono-Zn(II)-Sfh-I imipenem product complex (solid lines), and corresponding curve fits (open symbols). Supplementary Table 4 shows detailed EXAFS curve fitting results. (**d**) Comparison of EXAFS Fourier transforms for resting mono-Zn(II)-Sfh-I (gray), and the 10 ms ES species (black).

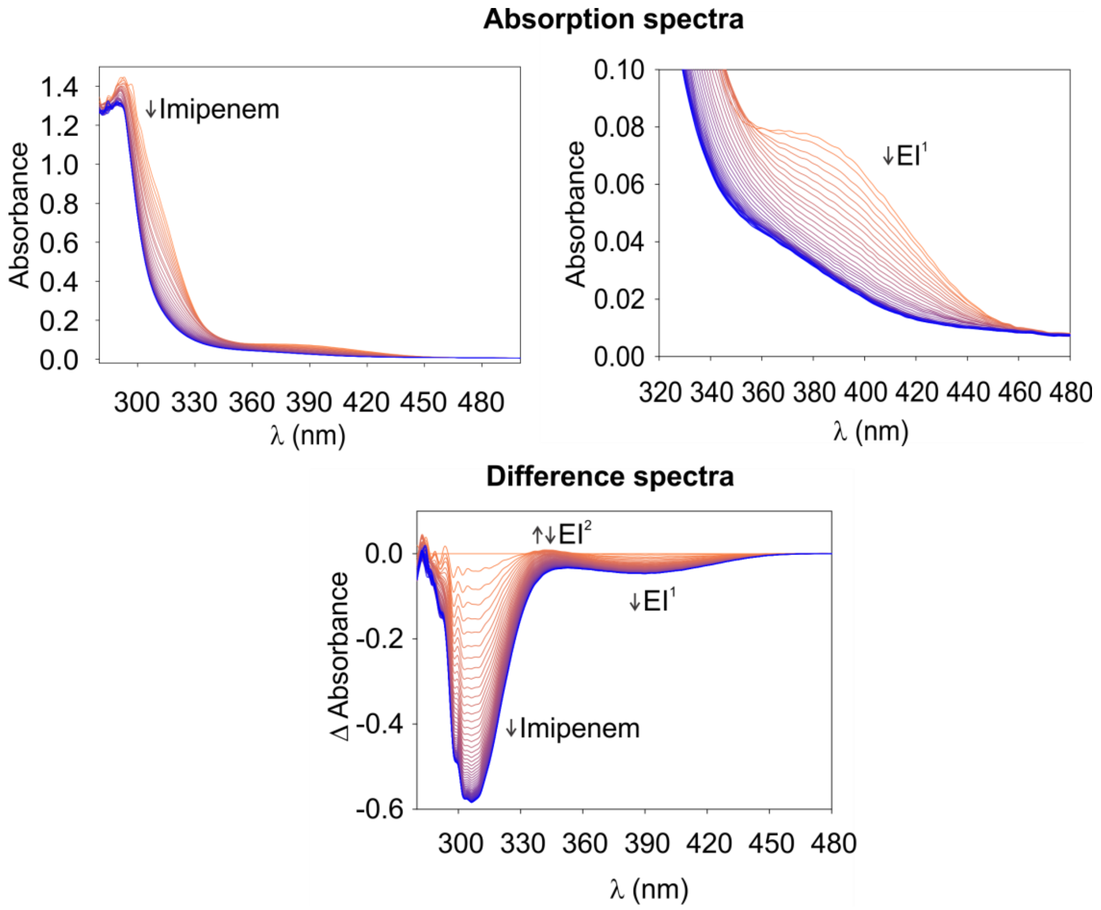

Supplementary Figure 12 | Electronic absorption spectra of imipenem hydrolysis catalyzed by bi-Zn(II)-NDM-1 under pre-steady state conditions. Hydrolysis of 150 uM imipenem by 100 μM bi-Zn(II)-NDM-1 detected employing a stopped-flow mixer coupled to a photodiode array detector. 1000 spectra were recorded in 10 s, with a logarithmic time base. The measurement was performed in 100 mM Hepes, pH 7.5, 200 mM NaCl and 300 μM $ZnSO_4$ at 6 ºC. Upper panels: sequence of electronic spectra up to 0.07 s of reaction. Lower panel: sequence of differential spectra corresponding to the data presented in the upper panel (obtained by subtraction of the first spectrum recorded). Two transient species were detected: EI^1 and EI^2. The accumulation of EI^1 was evidenced in the early steps of the reaction by its absorbance at 390 nm. EI^2 accumulated later, showing in the differential spectra a λ_{max} at 343 nm. The reaction progresses from orange to blue spectra.

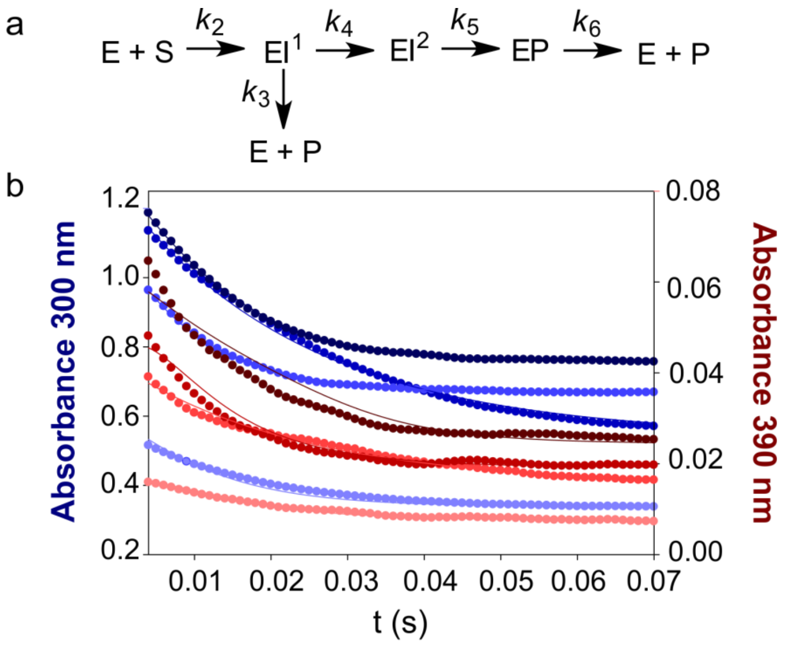

c

k_2	$(17 \pm 1)\ s^{-1}\ \mu M^{-1}$
k_3	$(48.7 \pm 0.6)\ s^{-1}$
k_4	$(42.8 \pm 0.6)\ s^{-1}$
k_5	$(0.0158 \pm 0.0008)\ s^{-1}$
k_6	$(0.077 \pm 0.007)\ s^{-1}$
$\varepsilon_{EI1\ 300\ nm}$	$(10{,}400 \pm 100)\ M^{-1}\ cm^{-1}$
$\varepsilon_{EI2\ 300\ nm}$	$(7{,}000 \pm 2{,}000)\ M^{-1}\ cm^{-1}$
$\varepsilon_{EI1\ 390\ nm}$	$(600 \pm 100)\ M^{-1}\ cm^{-1}$
$\varepsilon_{EI2\ 390\ nm}$	$(260 \pm 50)\ M^{-1}\ cm^{-1}$

Supplementary Figure 13 | Global fit of traces of imipenem hydrolysis catalyzed by bi-Zn(II)-NDM-1 under pre-steady state conditions. (**a**) Scheme that describes the kinetic model which allowed the best fit of the experimental data after an analysis similar to that described in Supplementary Fig. 2. It includes two transient and productive species (EI^1 and EI^2) and the complex of the enzyme (E) with product (EP). (**b**) Evolution of the absorbance at 300 nm (blue) and 390 nm (red) upon the reaction of imipenem and di-Zn(II)-NDM-1. From light to dark tones the initial concentrations of enzyme and substrate were: 50 µM imipenem and 47.5 µM di-Zn(II)-NDM-1; 150 µM imipenem and 47.5 µM di-Zn(II)-NDM-1; 100 µM imipenem and 95 µM di-Zn(II)-NDM-1 and 150 µM imipenem and 95 µM di-Zn(II)-NDM-1. The measurements were performed in 100 mM Hepes, pH 7.5, 200 mM NaCl and 300 µM $ZnSO_4$ at 6 ºC. The results of the fits to the kinetic model in (a) are shown as lines overlaid with the experimental data (dots). Analysis was made with data obtained through 0.5 s of reaction. (**c**) Parameters derived from the fit with the program DynaFit[1]. Fixed absorption coefficients are detailed in Materials and Methods.

a

b

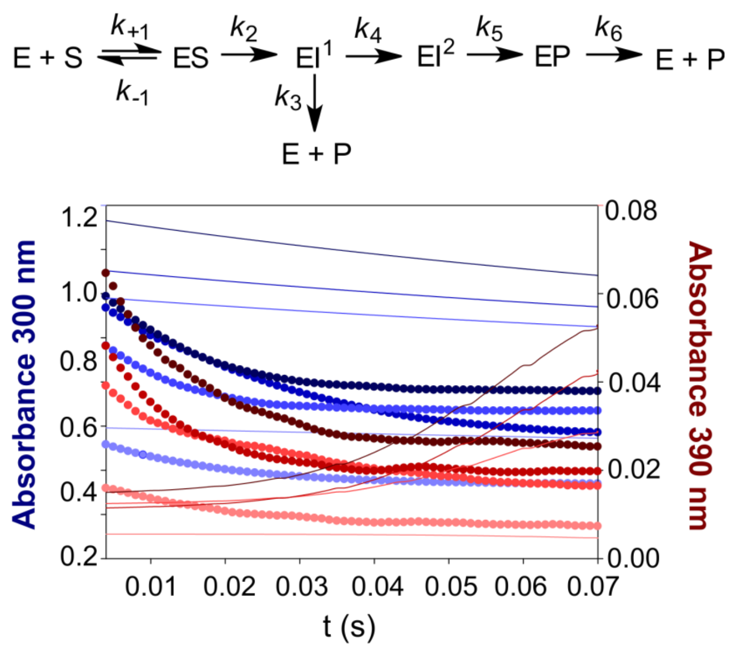

c

k_{+1}	ND
k_{-1}	ND
k_2	ND
k_3	ND
k_4	ND
k_5	ND
k_6	ND
$\varepsilon_{EI1\ 300\ nm}$	ND
$\varepsilon_{EI2\ 300\ nm}$	ND
$\varepsilon_{EI1\ 390\ nm}$	ND
$\varepsilon_{EI2\ 390\ nm}$	ND

Supplementary Figure 14 | Fitting traces of imipenem hydrolysis catalyzed by bi-Zn(II)-NDM-1 under pre-steady state conditions; including an ES complex. (**a**) Scheme that describes the kinetic model which allowed the best fit of the experimental data (Supplementary Fig. 13a) but incluiding an ES complex. (**b**) Evolution of the absorbance at 300 nm (blue) and 390 nm (red) upon the reaction of imipenem and di-Zn(II)-NDM-1. From light to dark tones the initial concentrations of enzyme and substrate were: 50 µM imipenem and 47.5 µM di-Zn(II)-NDM-1; 150 µM imipenem and 47.5 µM di-Zn(II)-NDM-1; 100 µM imipenem and 95 µM di-Zn(II)-NDM-1 and 150 µM imipenem and 95 µM di-Zn(II)-NDM-1. The measurements were performed in 100 mM Hepes, pH 7.5, 200 mM NaCl and 300 µM $ZnSO_4$ at 6 ºC. The results of the fits to the kinetic model in (a) are shown as lines overlaid with the experimental data (dots). Analysis was made with data obtained through 0.5 s of reaction. (**c**) Parameters derived from the fit with the program DynaFit[1]. Fixed absorption coefficients are detailed in Materials and Methods. ND: not defined value.

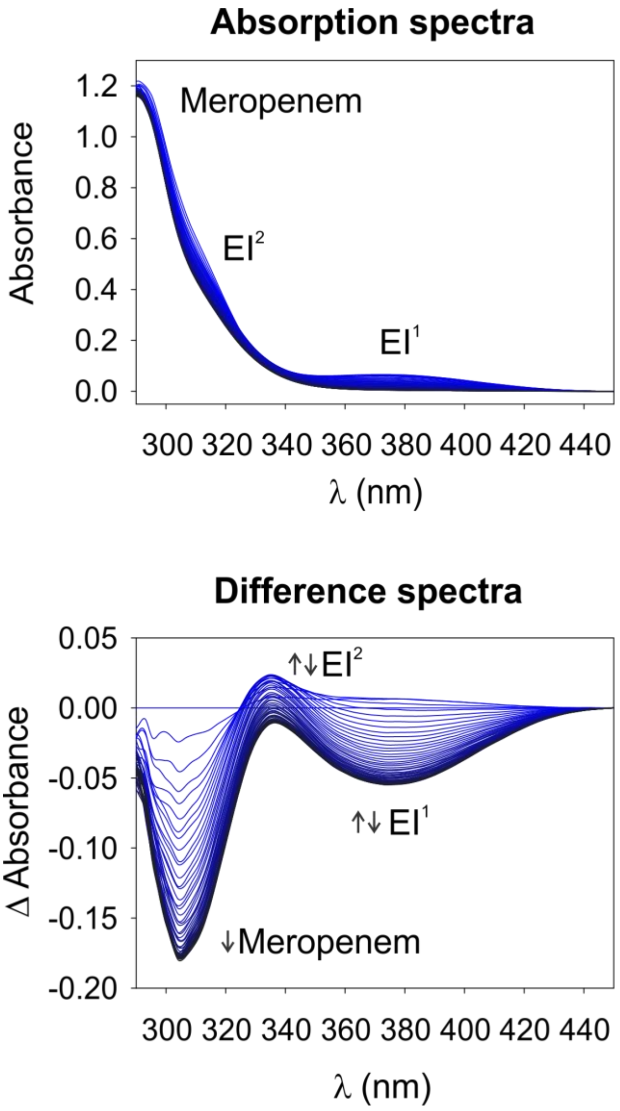

Supplementary Figure 15 | Electronic absorption spectra of meropenem hydrolysis catalyzed by bi-Zn(II)-NDM-1 under pre-steady state conditions. Hydrolysis of 100 uM imipenem by 100 μM bi-Zn(II)-NDM-1 detected employing a stopped-flow mixer coupled to a photodiode array detector. 1000 spectra were recorded in 10 s, with a logarithmic time base. The measurement was performed in 100 mM Hepes, pH 7.5, 200 mM NaCl and 300 μM $ZnSO_4$ at 6 ºC. Upper panel: sequence of electronic spectra up to 0.05 s of reaction. Lower panel: sequence of differential spectra corresponding to the data presented in the upper panel (obtained by subtraction of the first spectrum recorded). As in imipenem hydrolysis, two transient species were detected corresponding to EI^1 and EI^2. The accumulation of EI^1 was evidenced in the early steps of the reaction by its absorbance at 375 nm and EI^2 accumulated later, showing a maximum of absorption in the differential spectra at 335 nm. The reaction progresses from blue to black spectra.

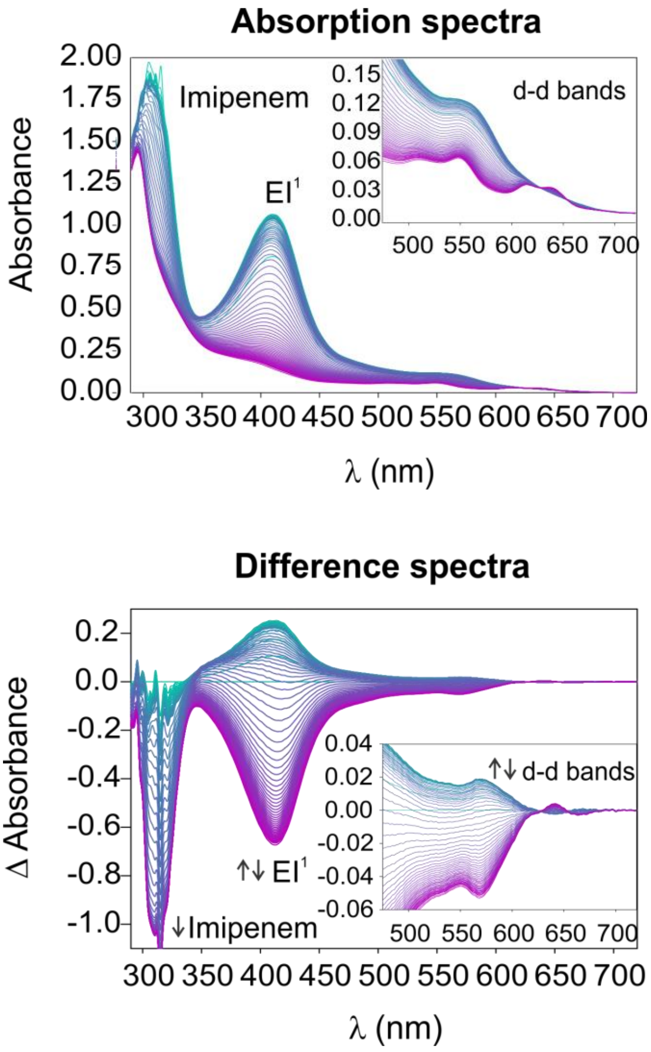

Supplementary Figure 16 | Electronic absorption spectra of imipenem hydrolysis catalyzed by bi-Co(II)-NDM-1 under pre-steady state conditions. Hydrolysis of 450 µM imipenem by 150 µM bi-Co(II)-NDM-1 detected employing a stopped-flow mixer coupled to a photodiode array detector. 1000 spectra were recorded in 10 s, with a logarithmic time base. The measurement was performed in 100 mM Hepes, pH 7.5, 200 mM NaCl and 2 equivalents of $CoSO_4$ at 6 ºC. Upper panel: sequence of electronic spectra up to 0.7 s of reaction. Lower panel: sequence of differential spectra corresponding to the data presented in the upper panel (obtained by subtraction of the first spectrum recorded). Changes in the d-d bands were clearly observed (450-700 nm) and the band at 412 nm revealed the accumulation and later decay of an intermediate (EI^1). The band at higher energies (343 nm) partially overlaps with the absorption of imipenem. The reaction progresses from green to purple spectra.

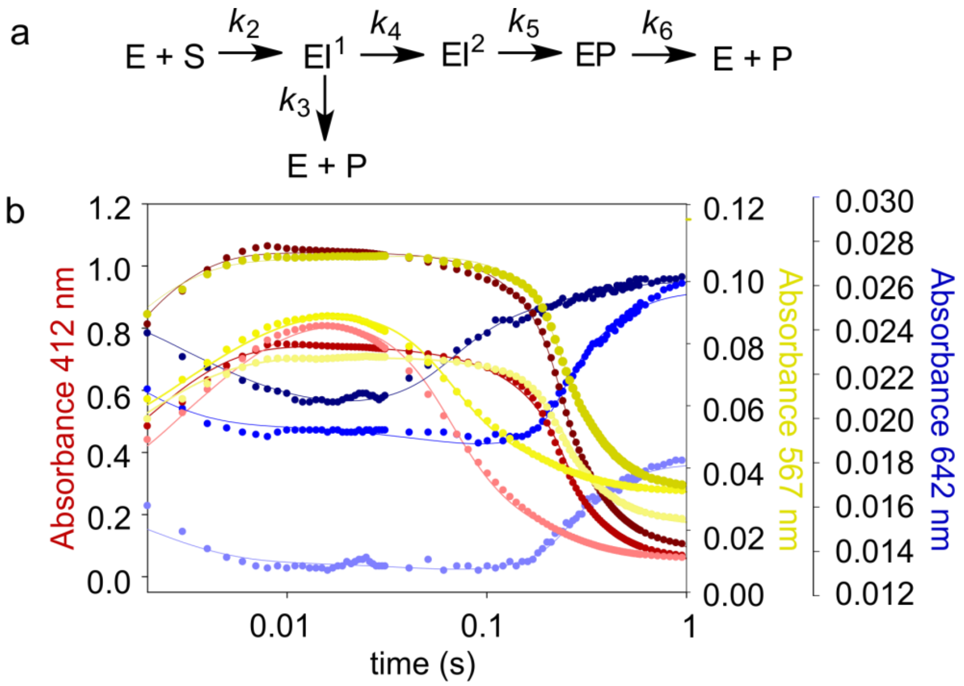

c

k_2	(1.98 ± 0.02) s^{-1} μM^{-1}	$\varepsilon_{EP\ 412\ nm}$	(800 ± 100) M^{-1} cm^{-1}
k_3	(24.0 ± 0.2) s^{-1}	$\varepsilon_{EI1\ 567\ nm}$	(980 ± 50) M^{-1} cm^{-1}
k_4	(4.17 ± 0.08) s^{-1}	$\varepsilon_{EI2\ 567\ nm}$	(1,160 ± 30) M^{-1} cm^{-1}
k_5	(5.0 ± 0.1) s^{-1}	$\varepsilon_{EP\ 567\ nm}$	(270 ± 30) M^{-1} cm^{-1}
k_6	0.1 s^{-1} (fixed)	$\varepsilon_{EI1\ 642\ nm}$	(174 ± 2) M^{-1} cm^{-1}
$\varepsilon_{EI1\ 412\ nm}$	(9,700 ± 600) M^{-1} cm^{-1}	$\varepsilon_{EI2\ 642\ nm}$	(125 ± 8) M^{-1} cm^{-1}
$\varepsilon_{EI2\ 412\ nm}$	(10,200 ± 300) M^{-1} cm^{-1}	$\varepsilon_{EP\ 642\ nm}$	(220 ± 13) M^{-1} cm^{-1}

Supplementary Figure 17 | Global fit of traces of imipenem hydrolysis catalyzed by bi-Co(II)-NDM-1 under pre-steady state conditions. (**a**) Scheme that describes the kinetic model which allowed the best fit of the experimental data after an analysis similar to that described in Supplementary Fig. 2. It includes two transient and productive species (EI^1 and EI^2) and the complex of the enzyme (E) with product (EP). This mechanism resembles the same presented for bi-Zn(II)-NDM-1. (**b**) Evolution of the absorbance at 412 nm (red dots), 567 nm (yellow dots) and 642 nm (blue dots) upon the reaction of imipenem and di-Co(II)-NDM-1. From light to dark tones the initial concentrations of enzyme and substrate were: 300 μM imipenem and 100 μM enzyme; 150 μM imipenem and 150 μM enzyme and 450 μM imipenem and 150 μM enzyme. The measurements were performed in 100 mM Hepes, pH 7.5, 200 mM NaCl and 2 equivalents of $CoSO_4$ at 6 ºC. The results of the fits to the kinetic model in (a) are shown as lines overlaid with the experimental data (dots). Analysis was made with data obtained through 1 s of reaction. (**c**) Parameters derived from the fit with the program DynaFit[1]. Fixed absorption coefficients are detailed in Materials and Methods.

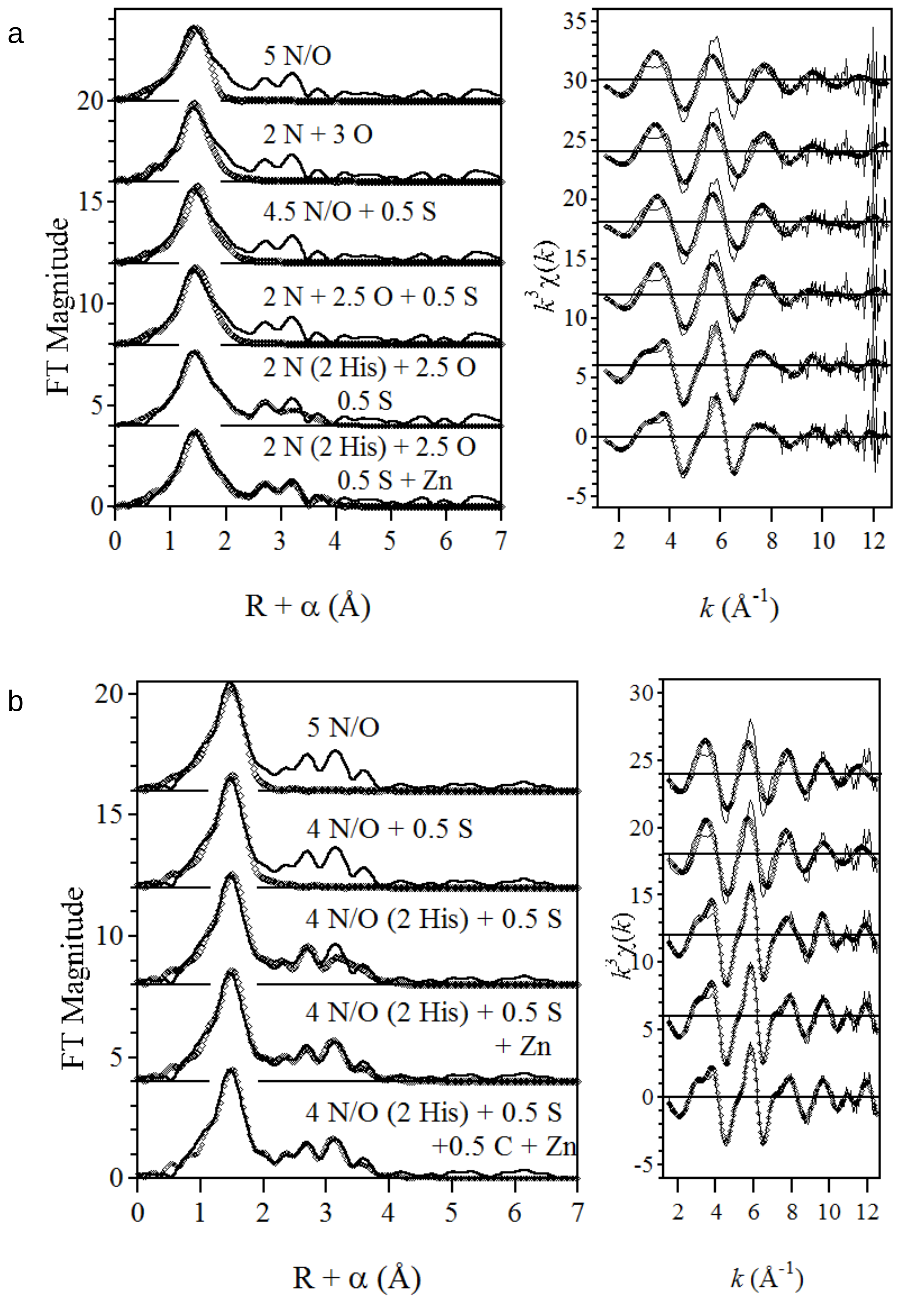

Supplementary Figure 18 | Fourier transforms of k^3-weighted EXAFS for bi-Zn(II)-BcII reaction.

c

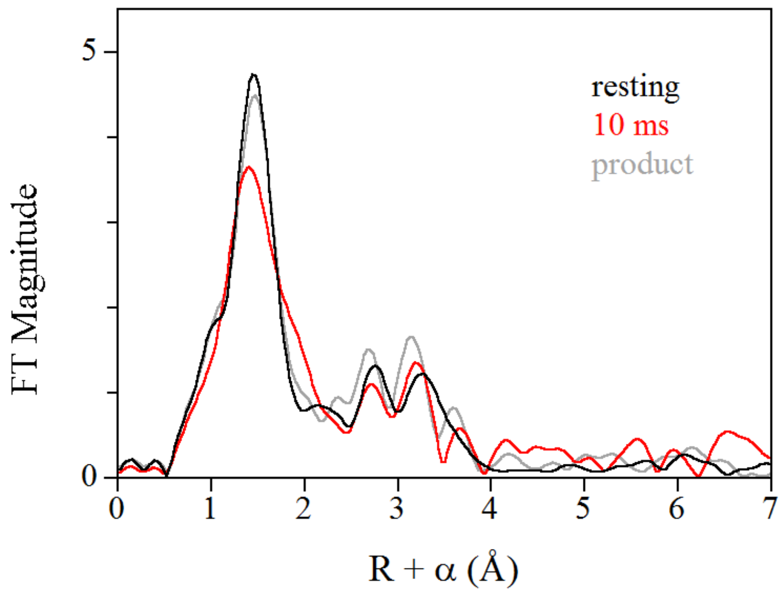

Supplementary Figure 18 cont. | Fourier transforms of k^3-weighted EXAFS for bi-Zn(II)-BcII reaction. (**a**) Fourier transforms (left) of k^3-weighted EXAFS (right) for bi-Zn(II)-BcII freeze-quenched after 10 ms of reaction with imipenem (solid lines), and corresponding curve fits (open symbols). (**b**) Fourier transforms (left) of k^3-weighted EXAFS (right) for bi-Zn(II)-BcII-imipenem product complex (solid lines), and corresponding curve fits (open symbols). Supplementary Table 5 shows detailed EXAFS curve fitting results. (**c**) Comparison of EXAFS Fourier transforms for resting bi-Zn(II)-BcII (black), the 10 ms intermediate (red), and the enzyme-product complex (grey).

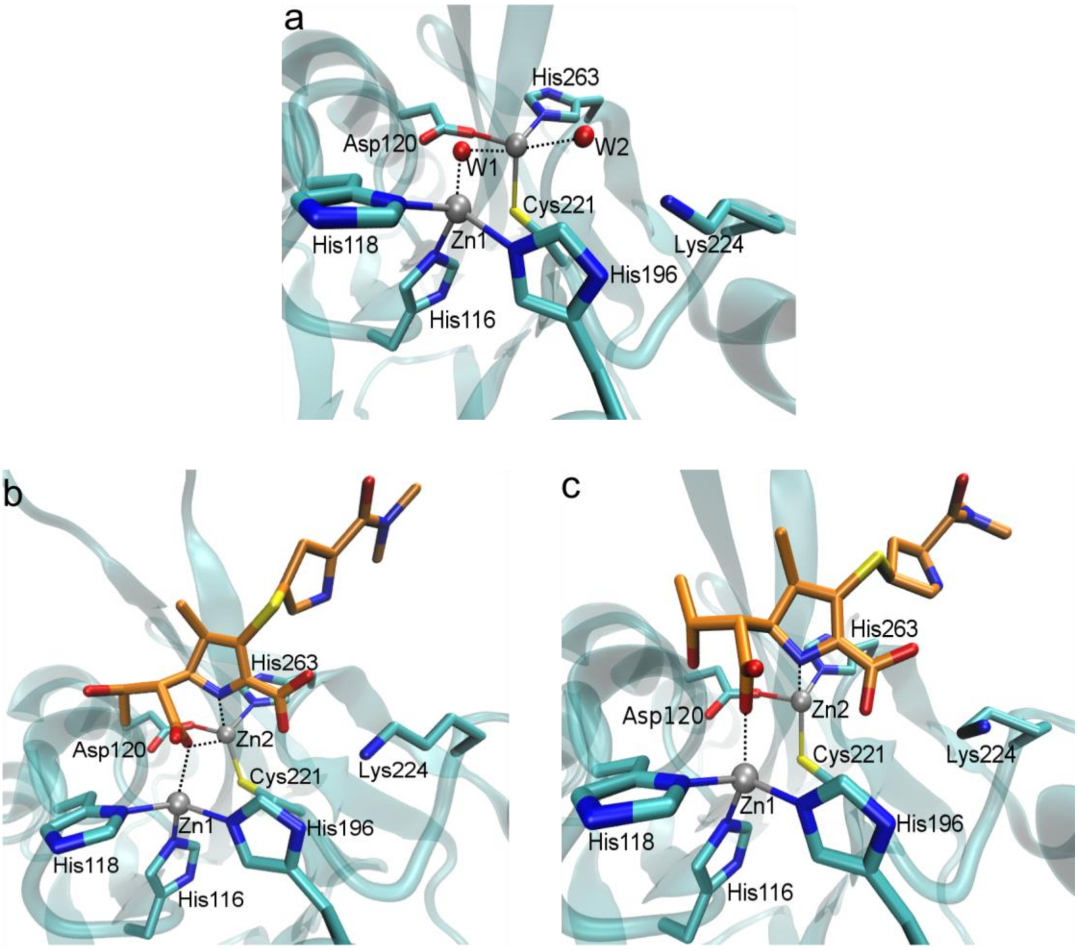

Supplementary Figure 19 | Crystal structures of bi-Zn(II)-NDM-1 and hydrolyzed meropenem. *K. pneumoniae* bi-Zn(II)-NDM-1: free enzyme structure PDB 3zr9 (**a**) and enzyme in complex with hydrolysed meropenem PDB 4rbs (**b**) and 4eyl (**c**). The protein structure is depicted with the conventional liquorice colours (C cyan, H white, O red, N blue and S yellow). Zn(II) ions are shown as grey spheres. Carbon atoms of the antibiotic derived ligand are shown in orange. Coordination bonds of the amino acid residues to Zn(II) are shown with solid lines and interactions with the antibiotic derived ligand are shown with dotted lines.

.

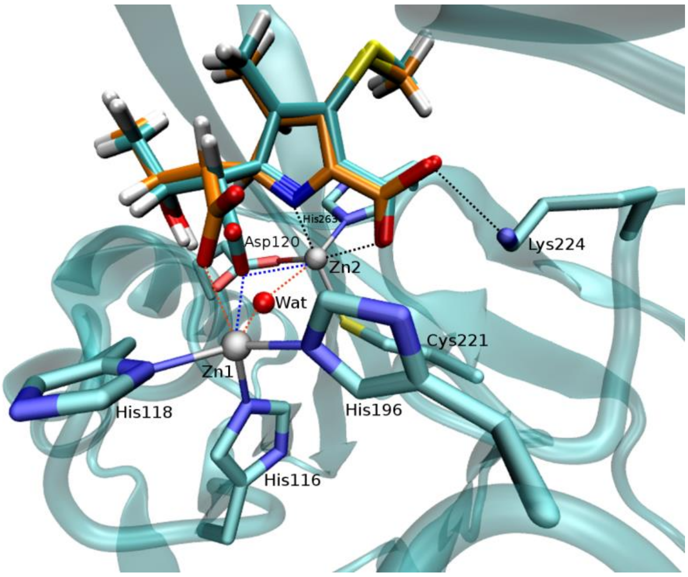

Supplementary Figure 20 | Superimposition of the proposed structures of EI^1 and EI^2. The quantum protein subsystem is depicted with the conventional liquorice colours (C cyan, H white, O red, N blue and S yellow). Zn(II) ions are shown as grey spheres. Carbons of the antibiotic derived ligand in EI^1 are shown in orange and in EI^2 in cyan. Coordination bonds of amino acid residues to Zn(II) are shown with solid lines, coordination bonds of groups in the antibiotic-derived-ligand to Zn(II) and hydrogen-bonds to amino acid groups are shown as dotted lines.

a

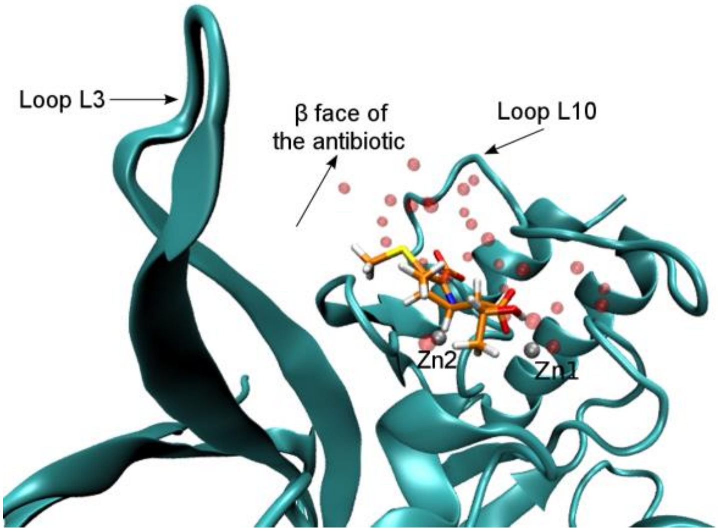

b

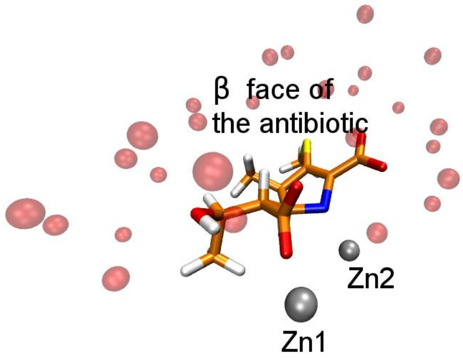

Supplementary Figure 21 | Water distribution on the active site of bi-Zn(II)-NDM-1 in complex with hydrolysed meropenem. (**a**) Waters located at 5 Å of C-2 in a snapshot of the molecular dynamics of the EI^2 formed during NDM-1 and meropenem reaction. (**b**) Same as figure (a) but rotated around the vertical axis and without protein scaffold.

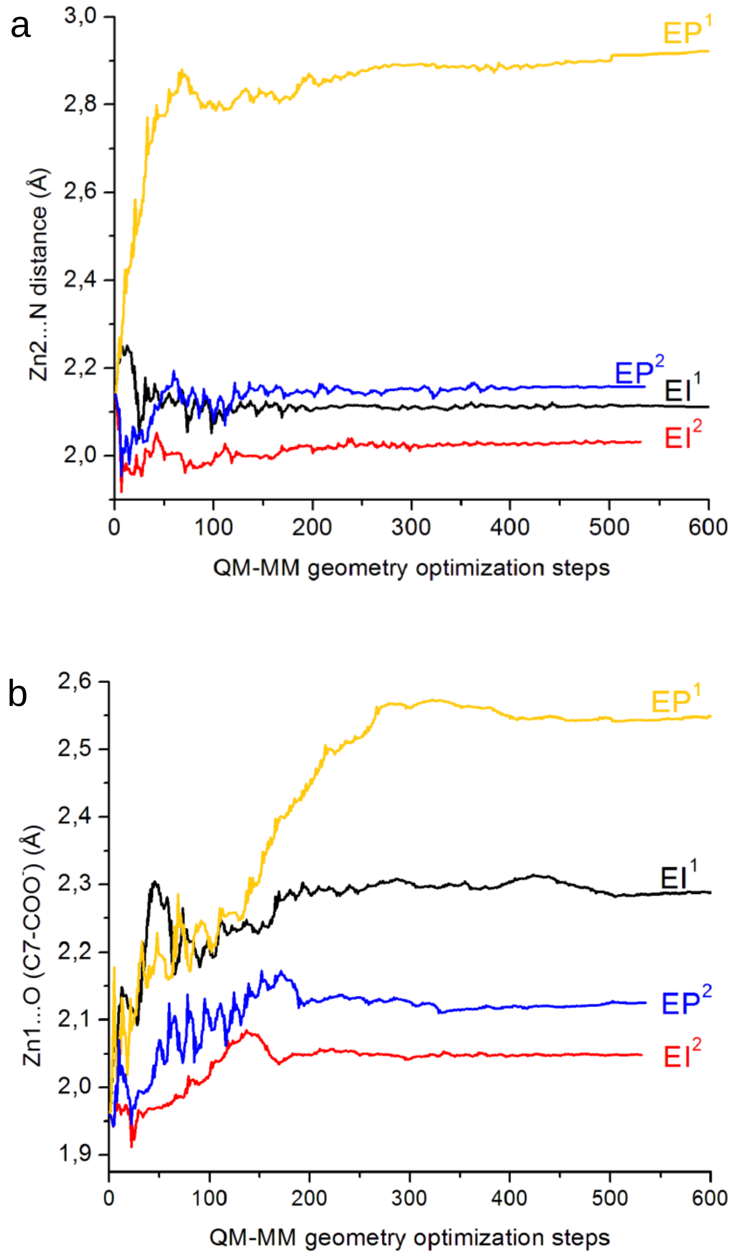

Supplementary Figure 22 | Evolution of the distance during the QM-MM geometry optimizations. (**a**) Evolution of the distance (in Å) between Zn_2 and the nitrogen atom of EI^1 (in black), EI^2 (in red), EP^1 (in yellow) or EP^2 (in blue) during the QM-MM geometry optimizations. (**b**) Evolution of the distance (in Å) between Zn_1 and one of the oxygen atoms of the carboxylate moiety (C7-COO^-) of EI^1 (in black), EI^2 (in red), EP^1 (in yellow) or EP^2 (in blue) during the QM-MM geometry optimizations.

Supplementary Table 1 | λ_{max} of intermediate species observed during hydrolysis of carbapenems by MβLs monitored by stopped flow techniques and based on the difference spectra

	**EI^1**	**EI^2**
Mono-Zn(II)-GOB-18 – Imipenem	ND	340 nm*
Mono-Co(II)-GOB-18 – Imipenem	ND	340 nm
Mono-Zn(II)-Sfh-I – Imipenem	390 nm	340 nm
Bi-Zn(II)-NDM-1 – Imipenem	390 nm	343 nm
Bi-Co(II)-NDM-1 – Imipenem	412 nm	ND
Bi-Zn(II)-NDM-1 – Meropenem	375 nm	336 nm
Bi-Zn(II)-BcII – Imipenem[2]	380 nm	330 nm
Co-Zn(II)-BcII – Imipenem[2]	407 nm	ND

ND: Not detected.

(*) For mono-Zn(II)-GOB-18 the spectrum of EI^2 is presented in Supplementary Figure 3.c. with λ_{max}=300 nm

Supplementary Table 2 | Fourier transforms of k3-weighted EXAFS for mono-Zn(II)-GOB-18 reaction

Fit	Model	*Zn-O*	*Zn-N*	$Zn\text{-}C_{CO_2^-}$	*Zn-His* [b]	R_f [c]	R_u [c]
S.4a-1	5 N/O		2.07 (6.4)			84	321
S.4a-2	3 N + 2 O	1.97 (0.9)	2.13 (4.8)			33	286
S.4a-3	3 N (2 His) + 2 O	1.96 (2.9)	2.14 (11)		2.94 (2.6) 3.12 (0.1) 4.17 (17) 4.44 (15)	223	237
S.4a-4	3 N (2 His) + 2 O + 1 C	1.96 (4.3)	2.14 (9.6)	2.50 (1.6)	2.95 (1.3) 3.11 (6.1) 4.18 (13) 4.43 (19)	183	200
S.4b-1	5 N/O		2.08 (9.2)			68	310
S.4b-2	3 N + 2 O	1.99 (1.9)	2.15 (1.8)			33	281
S.4b-3	3 N (2 His) + 2 O	1.99 (1.1)	2.14 (3.2)		2.86 (5.0) 2.94 (2.1) 3.93 (3.9) 4.50 (20)	96	237
S.4b-4	3 N (2 His) + 2 O + 1 C	1.99 (1.3)	2.15 (1.9)	2.65 (0.6)	2.84 (4.9) 3.19 (1.6) 4.02 (7.3) 4.50 (21)	72	182

The table shows detailed EXAFS curve fitting results for GOB freeze-quenched after 10 ms of reaction with imipenem[a] (S.4a) and curve fitting results for the GOB-imipenem product complex[a] (S.4b). Graphics are shown in Supplementary Figure 4.

Notes:

[a] Distances (Å) and disorder parameters (in parentheses, σ^2 (10^{-3} Å^2)) shown derive from integer or half-integer coordination number fits to filtered EXAFS data [k = 1.5-12.5 Å^{-1}; R = 0.7-2.2 Å (fits 1-2) or 0.1-4.2 Å (fits 3-4)].

[b] Multiple scattering paths represent combined paths, as described previously (see Materials and Methods).

[c] Goodness of fit (R_f for fits to filtered data; R_u for fits to unfiltered data) defined as 1000* $\frac{\sum_{i=1}^{N}\left\{\left[\mathrm{Re}(\chi_{i,obs})^2+\mathrm{Im}(\chi_{i,obs})^2\right]-\left[\mathrm{Re}(\chi_{i,calc})^2+\mathrm{Im}(\chi_{i,calc})^2\right]\right\}}{\sum_{i=1}^{N}\left\{\left[\mathrm{Re}(\chi_{i,obs})^2+\mathrm{Im}(\chi_{i,obs})^2\right]\right\}}$, where N is the number of data points.

Supplementary Table 3 | Steady-state kinetic parameters for the hydrolysis of imipenem

	K_m (µM)	k_{cat} (s^{-1})	k_{cat}/K_m ($s^{-1}µM^{-1}$)
**Mono-Zn-GOB-18**[3]	28±9	34±4	1.3±0.4
**Mono-Co-GOB-18**[3]	26±6	3.3±0.2	0.13±0.03
**Mono-Zn-SfhI**	52±5	83±3	1.6±0.2
**Mono-Co-SfhI**	55±3	23.6±0.4	0.43±0.03
**Bi-Zn-NDM-1**	160±40	350±20	2.19±0.7
**Bi-Co-NDM-1**	80±30	89±5	1.1±0.5

Kinetic parameters were derived from nonlinear fit of the Michaelis-Menten equation to initial rate measurements. The reaction medium was 10 mM Hepes, pH 7.5, 200 mM NaCl, 20 µM $ZnSO_4$ or $CoSO_4$, 50 µg/mL BSA, at 30 °C.

Supplementary Table 4 | Fourier transforms of k^3-weighted EXAFS for mono-Zn(II)-Sfh-I reaction

Fit	Model	*Zn-N/O*	*Zn-S*	*Zn-His* [b]	R_f [c]	R_u [c]
S.11a-1	6 N/O	2.05 (7.7)			159	191
S.11a-2	3 N/O + 1 S	2.02 (5.2)	2.28 (3.6)		11	97
S.11a-3	3 N/O (1 His) + 1 S	2.02 (4.8)	2.29 (3.5)	2.82 (11) 3.19 (0.1) 4.11 (17) 4.47 (12)	45	52
S.11b-1	6 N/O	2.06 (8.6)			200	350
S.11b-2	3 N/O + 1 S	2.02 (3.7)	2.28 (3.3)		28	253
S.11b-3	3 N/O (1 His) + 1 S	2.02 (2.3)	2.29 (3.0)	2.84 (11) 3.61 (0.1) 4.26 (2.1) 4.37 (1.1)	196	209
S11c-1	6 N/O	2.05 (8.2)			117	176
S.11c-2	3 N/O + 1 S	2.01 (3.3)	2.28 (3.9)		13	97
S.11c-3	3 N/O (1 His) + 1 S	2.02 (3.1)	2.29 (4.3)	2.82 (6.8) 3.17 (0.1) 4.08 (14) 4.47 (14)	55	67

The table shows detailed EXAFS curve fitting results for resting mono-Zn(II)-Sfh-I[a] (S.11a), curve fitting results for mono-Zn(II)-Sfh-I freeze-quenched after 10 ms of reaction with imipenem[a] (S. 11b) and curve fitting results for mono-Zn(II)-Sfh-I imipenem product complex[a] (S. 11c). Graphics are shown in Supplementary Figure 11.

Notes:

[a] Distances (Å) and disorder parameters (in parentheses, σ^2 (10^{-3} Å^2)) shown derive from integer or half-integer coordination number fits to filtered EXAFS data [k = 1.5-12.5 Å^{-1}; R = 0.7-2.2 Å (fits 1-2) or 0.1-4.2 Å (fits 3-4)].

[b] Multiple scattering paths represent combined paths, as described previously (see Materials and Methods).

[c] Goodness of fit (R_f for fits to filtered data; R_u for fits to unfiltered data) defined as 1000* $\frac{\sum_{i=1}^{N}\left\{\left[\mathrm{Re}(\chi_{i,obs})^2+\mathrm{Im}(\chi_{i,obs})^2\right]-\left[\mathrm{Re}(\chi_{i,calc})^2+\mathrm{Im}(\chi_{i,calc})^2\right]\right\}}{\sum_{i=1}^{N}\left\{\left[\mathrm{Re}(\chi_{i,obs})^2+\mathrm{Im}(\chi_{i,obs})^2\right]\right\}}$, where N is the number of data points.

Supplementary Table 5 | Fourier transforms of k^3-weighted EXAFS for bi-Zn(II)-BcII reaction

Fit	Model	*Zn-N/O*	*Zn-S*	*Zn-His* [b]	*Zn-Zn*	R_f [c]	R_u [c]
S.18a-1	5 N/O	2.06 (8.5)				187	386
S.18a-2	2 N + 3 O	1.97 (2.5)<br>2.14 (5.3)				114	337
S.18a-3	4.5 N/O + 0.5 S	2.04 (4.1)	2.29<br>(2.9)			88	316
S.18a-4	2 N + 2.5 O + 0.5 S	1.97 (3.6)<br>2.15 (4.9)	2.30<br>(3.4)			69	307
S.18a-5	2 N (2 His) + 2.5 O + 0.5 S	1.97 (3.7)<br>2.12 (5.4)	2.30<br>(4.1)	2.91 (4.8) 3.13 (1.2)<br>4.18 (13) 4.43 (16)		102	188
S.18a-6	2 N (2 His) + 2.5 O + 0.5 S + Zn-Zn [d]	1.97 (3.2)<br>2.13 (6.2)	2.30<br>(3.3)	2.91 (4.9) 3.13 (1.2)<br>4.20 (13) 4.43 (16)	3.82<br>(5.3)	36	123
S.18b-1	5 N/O	2.03 (7.8)				89	243
S.18b-2	4 N/O + 0.5 S	2.02 (5.6)	2.29<br>(4.5)			48	225
S.18b-3	4 N/O (2 His) + 0.5 S	2.02 (5.9)	2.28<br>(5.6)	2.93 (2.9) 3.15 (1.0)<br>4.14 (18) 4.44 (24)		95	76
S.18b-4	4 N/O (2 His) + 0.5 S + Zn-Zn	2.02 (6.0)	2.28<br>(6.0)	2.94 (3.3) 3.13 (0.8)<br>4.16 (17) 4.44 (15)	3.51<br>(7.9)	34	57
S.18b-5	4 N/O (2 His) + 0.5 S + 0.5 C + Zn-Zn [d]	2.02 (6.0)	2.30<br>(7.3)	2.94 (3.3) 3.13 (0.9)<br>4.17 (14) 4.44 (21)	3.51<br>(8.1)	26	44

The table shows detailed EXAFS curve fitting results for bi-Zn(II)-BcII freeze-quenched after 10 ms of reaction with imipenem[a] (S.18a) and fitting results for the bi-Zn(II)-BcII-imipenem product complex[a] (S.18b). Graphics are shown in Supplementary Figure 18.

Notes:

[a] Distances (Å) and disorder parameters (in parentheses, σ^2 (10^{-3} Å^2)) shown derive from integer or half-integer coordination number fits to filtered EXAFS data [k = 1.5-12.5 Å^{-1}; R = 0.7-2.2 Å (fits 1-2) or 0.1-4.2 Å (fits 3-4)].

[b] Multiple scattering paths represent combined paths, as described previously (see Materials and Methods).

[c] Goodness of fit (R_f for fits to filtered data; R_u for fits to unfiltered data) defined as 1000*

$$\frac{\sum_{i=1}^{N}\left\{\left[\mathrm{Re}(\chi_{i,obs})^2+\mathrm{Im}(\chi_{i,obs})^2\right]-\left[\mathrm{Re}(\chi_{i,calc})^2+\mathrm{Im}(\chi_{i,calc})^2\right]\right\}}{\sum_{i=1}^{N}\left\{\left[\mathrm{Re}(\chi_{i,obs})^2+\mathrm{Im}(\chi_{i,obs})^2\right]\right\}},$$

where N is the number of data points.

[d] Inclusion of two Zn-Zn populations yielded a maximum reduction in fit residual to 29, with 0.1 Zn at 3.51 Å and 0.9 Zn at 3.82 Å.

Supplementary Table 6 | Relevant distances (Å) and angles (°) of crystal structure (pdb code 4RBS) and QM-MM optimized structures

Distance	4RBS chain A / B		EI[1] (from 4RBS)	EI[2] (from 4RBS)	4EYL chain A / B		EI[1] (from 4EYL)	EI[2] (from 4EYL)
$Zn_1...Zn_2$	4.00	4.00	4.35	3.66	4.05	3.88	4.39	4.08
Zn_2-N	2.14	2.15	2.11	2.03	2.21	2.32	2.13	2.05
Zn_2...O (C3-COO)	2.99	3.10	2.35	2.70	3.02	2.89	3.39	3.29
Zn_1-O (C7-COO)	1.96	2.15	2.28	2.05	2.28	2.24	2.12	2.05
Zn_2...O (C7-COO)	3.27	2.99	3.95	2.46	2.46	2.67	3.43	2.81
C7...N	2.43	2.53	2.73	2.66	2.45	2.46	2.82	2.63
N-C3	1.33	1.33	1.37	1.37	1.29	1.30	1.36	1.36
C2-C3	1.33	1.34	1.41	1.42	1.35	1.34	1.42	1.42
Zn_1...O (water)			2.36				2.63	
Zn_2...O (water)			2.30				2.29	

Supplementary Table 7 | Mulliken charges of QM - MM optimized structures

	**EI[1] (starting from 4EYL)**	**EI[1] (starting from 4RBS)**	**EI[2] (starting from 4EYL)**	**EI[2] (starting from 4RBS)**
Zn_1	0.796	0.778	0.754	0.758
Zn_2	0.830	0.834	0.752	0.798
N (meropenem)	-0.172	-0.178	-0.154	-0.130
O (water)	0.064	-0.054		
C1	0.222	0.212	0.22	0.220
C2	0.006	0.012	0.010	-0.026
C3	-0.036	-0.048	-0.034	-0.058
C5	0.140	0.142	0.144	0.134
S	-0.378	-0.326	-0.41	-0.330

Supplementary Table 8 | Relevant distances (Å) and angles (°) of crystal structure (pdb code 4RBS) and QM-MM optimized structures

**Distance**	**4RBS chain A / B**		**EI[1]**	**EI[2]**	**EP[1]**	**EP[2]**
Zn_1...Zn_2	4.00	4.00	4.35	3.66	4.10	3.91
Zn2-N	2.14	2.15	2.11	2.03	2.94	2.19
Zn_2...O (C3-COO)	2.99	3.10	2.35	2.70	2.47	2.63
Zn_1-O (C7-COO)	1.96	2.15	2.28	2.05	2.65	2.14
Zn_2...O (C7-COO)	3.27	2.99	3.95	2.46	4.36	2.39
C7...N	2.43	2.53	2.73	2.66	2.71	2.65
N-C3	1.33	1.33	1.37	1.37	1.38	1.30
C2-C3	1.33	1.34	1.41	1.42	1.40	1.54
Zn_1...O(water)			2.36		2.11	
Zn_2...O(water)			2.30		2.06	
**Angles**						
Zn_2-N-C3	111.1	115.3	113.2	124.3	105.4	125.9
Zn_2-N-C5	115.2	108.0	132.5	126.5	138.4	123.1
N-C3-C2	110.0	110.1	115.0	114.6	111.6	112.9
C3-C2-C1	110.7	110.6	105.3	105.6	108.3	100.7
C3-C2-S	127.2	127.8	130.3	129.8	130.5	120.1
Zn_1-Zn_2-N	75.0	79.9	79.8	98.7	72.8	94.5

Supplementary Table 9 | Comparison of relevant imipenem hydrolysis constants

	$\boldsymbol{k_3}$ (s^{-1})	$\boldsymbol{k_5}$ (s^{-1})	$\boldsymbol{k_4}$ (s^{-1})	**ES**
**Mono-Zn-GOB-18**	132	0.0812	69	Yes
**Mono-Co-GOB-18**	24	0.141	16	Yes
**Mono-Zn-SfhI**	19	0.09	5.8 / 0.22 (*)	Yes
**Bi-Zn-NDM-1**	48.7	0.0158	42.8	No
**Bi-Co-NDM-1**	24	5	4.17	No

k_3 is the protonation rate of EI^1, k_5 is the protonation rate of EI_2, and k_4 is the conversion rate of EI^1 to EI^2.

(*) This step is reversible in this case.

Supplementary References

1. Kuzmic, P. Program DYNAFIT for the analysis of enzyme kinetic data: application to HIV proteinase. *Anal Biochem* 237, 260-73 (1996).

2. Tioni, M.F. et al. Trapping and characterization of a reaction intermediate in carbapenem hydrolysis by B. cereus metallo-beta-lactamase. *J Am Chem Soc* 130, 15852-63 (2008).

3. Lisa, M.N., Hemmingsen, L. & Vila, A.J. Catalytic role of the metal ion in the metallo-beta-lactamase GOB. *J Biol Chem* 285, 4570-7 (2010).
